# Mid-pass whole genome sequencing enables biomedical genetic studies of diverse populations

**DOI:** 10.1186/s12864-021-07949-9

**Published:** 2021-11-01

**Authors:** Anne-Katrin Emde, Amanda Phipps-Green, Murray Cadzow, C. Scott Gallagher, Tanya J. Major, Marilyn E. Merriman, Ruth K. Topless, Riku Takei, Nicola Dalbeth, Rinki Murphy, Lisa K. Stamp, Janak de Zoysa, Philip L. Wilcox, Keolu Fox, Kaja A. Wasik, Tony R. Merriman, Stephane E. Castel

**Affiliations:** 1 Variant Bio Inc., Seattle, WA USA; 2 Department of Biochemistry, University of Otago, Dunedin, New Zealand; 3 Division of Clinical Immunology and Rheumatology, University of Alabama at Birmingham, Birmingham, AL USA; 4 Department of Medicine, University of Auckland, Auckland, New Zealand; 5 University of Otago Christchurch, Christchurch, New Zealand; 6 Department of Mathematics and Statistics, University of Otago, Dunedin, New Zealand; 7 Departments of Anthropology and Global Health, University of California, San Diego, CA USA

## Abstract

**Background:**

Historically, geneticists have relied on genotyping arrays and imputation to study human genetic variation. However, an underrepresentation of diverse populations has resulted in arrays that poorly capture global genetic variation, and a lack of reference panels. This has contributed to deepening global health disparities. Whole genome sequencing (WGS) better captures genetic variation but remains prohibitively expensive. Thus, we explored WGS at “mid-pass” 1-7x coverage.

**Results:**

Here, we developed and benchmarked methods for mid-pass sequencing. When applied to a population without an existing genomic reference panel, 4x mid-pass performed consistently well across ethnicities, with high recall (98%) and precision (97.5%).

**Conclusion:**

Compared to array data imputed into 1000 Genomes, mid-pass performed better across all metrics and identified novel population-specific variants with potential disease relevance. We hope our work will reduce financial barriers for geneticists from underrepresented populations to characterize their genomes prior to biomedical genetic applications.

**Supplementary Information:**

The online version contains supplementary material available at 10.1186/s12864-021-07949-9.

## Background

Over the past decade, population and statistical genetics have relied heavily on genotyping panels as an alternative to costly sequencing approaches for generating genome-wide datasets. Due to their sparse coverage, arrays require informed selection of variants a priori and reference panels for downstream imputation. Genomic analyses of array data have revolutionized understanding of human disease and population histories, but the focus has been predominantly on individuals of Western European ancestry [1–3]. When combined, people with Latin American, African, or Indigenous ancestries represent only 4% of all participants in published genome-wide association studies (GWAS) [4]. Underrepresentation of diverse populations has resulted in a lack of reference panels for imputation and insufficient optimization of variant panels to adequately capture genetic variation at a global scale [5, 6]. As a result patients from underrepresented populations receive less accurate diagnostic predictions, are often excluded from clinical trials that require genetic stratification, and can be unresponsive to therapeutics that have been optimized for individuals of European ancestry [7–10]. Additionally, current barriers-to-entry are significantly higher for Indigenous and minority geneticists to characterize their communities’ genomes in a manner that is best aligned with their cultural values [11]. Ultimately, the democratization of genomic technologies will require the costs of generating genome-wide datasets to be dramatically reduced [12].

Unlike array-based genotyping, whole genome sequencing (WGS) is better able to capture novel genetic variation. While the cost of WGS has been decreasing, it remains prohibitively expensive for all but the best-funded genomic studies. To address this, the concept of “low-pass” sequencing, where each position in the genome is covered by reads at a lower depth than the gold standard 30x, has been gaining traction as a cost-effective alternative to genotyping arrays for applications such as GWAS and polygenic scores [13–16]. To date, many low-pass approaches have been focused on genomic coverage levels of 1x and lower, bringing costs in line with arrays [17]. Even off-target data from whole exome studies has been shown to be sufficient [18]. However, at such low coverage levels, imputation into large pre-existing reference panels is still required, preventing applications of low-pass sequencing to studies with underrepresented populations.

While the 1000 Genomes Project reference panel has become popular for the study of diverse populations, it is limited in size and significant improvements have been demonstrated when using a more appropriately matched, larger panel [19–21]. More extensive panels exist but are still limited in diversity and poorly represent many populations [22, 23]. Importantly, access restrictions around genomic data will, at least in the foreseeable future, continue to make large external reference panels, which usually require data to be uploaded to a server, of limited practical use.

Given this limitation, we explored the utility of a self-contained approach that is independent of external reference panels and makes use of standard, well-established and well-maintained software packages for variant calling and imputation. We investigated the performance of WGS across different coverage values ranging from 1-7x, which we term “mid-pass”. This strategy is not without precedent, as mid-pass sequencing followed by within-cohort imputation has been applied in the context of global surveys of genetic diversity and population-based studies [19, 21, 24–28]. However, to our knowledge, there has not been widespread uptake of the approach, in part due to the lack of a comprehensive evaluation of the usefulness of current standard software packages across a number of relevant metrics such as cohort size and coverage levels.

To address this, we developed and benchmarked methods for mid-pass sequencing and applied them to identify genetic variation in a population that lacks an existing genomic reference panel. This is an essential step before genome-wide and targeted approaches to understanding genetic contributions to disease pathogenesis. First, we benchmarked the performance of cost-effective, low-pass library generation kits against more expensive high-pass kits at mid-pass coverage levels. Next, we developed an optimized bioinformatics pipeline around the widely-used GATK Best Practices [29] [v4.1.4] coupled with Beagle [30] for within-cohort variant calling and imputation to produce high quality individual-level genotype calls from mid-pass data. This is in contrast to approaches estimating only population allele frequencies [31]. We also assessed if combining mid- and high-pass data could improve the quality of genotype calls cohort-wide. Lastly, we applied our mid-pass approach to characterize genetic diversity in an underrepresented population and extensively benchmarked it against genotyping arrays. Our aim is to establish a framework for cost-effective studies that democratize genomic analyses by making the generation of genomic data more affordable and accessible.

## Results

### Low-pass optimized methods for high-throughput library preparation scale to intermediate coverage levels and produce consistent data

As a result of the increasing adoption of low-pass sequencing, library preparation kits that facilitate highly multiplexed processing of DNA samples for low coverage (≤1x) WGS are now commercially available. High-throughput processing is required for low-pass approaches, since many samples are sequenced simultaneously (on the same flow cell) as compared to gold standard 30x WGS. However, many kits that are designed for low-pass sequencing have not been comprehensively tested at an intermediate coverage level of 4x. Thus, we began by assessing if a commonly used library preparation kit designed for low-pass sequencing would be suitable at an intermediate coverage level. To compare library preparation methods quantitatively, we obtained DNA from 12 HapMap individuals (see Methods), generated 4 replicate libraries using the low-pass kit, and generated 2 replicate libraries using a standard high-pass WGS kit. While we sequenced low-pass libraries at target coverages of 1x (LP1) and 4x (LP4), high-pass libraries were sequenced at a target coverage of 4x (HP4).

First, we observed that the low-pass kit produced more consistent coverage across pooled libraries than the high-pass kit (Fig. S1a). This is particularly important in low-pass applications, as high variability in coverage at low depths may result in large disparities in genotyping quality across samples. Next, we compared read duplication rates across the two library types. Duplication rates were consistently higher for the low-pass kit across all coverage levels and increased proportionally with coverage. In contrast, duplication rates for the high-pass kit were consistent across the coverage ranges tested (Fig. S1b). Specifically, at target coverages of 1x and 4x, samples had median duplication rates of 10.8 and 17.2%, respectively, which were 1.5x and 2.4x greater than the high-pass kit. Next, we assessed genotype quality (GQ) scores derived from standard sample-level genotype calling (Fig. S1c). At 4x coverage, GQ scores derived from low-pass kit data did not significantly differ from GQ scores derived from high-pass kit data (*p* = 0.78, Wilcoxon rank sum test).

Next, we compared both overlap and concordance of genotype calls between replicates across coverage levels and kits. At 4x coverage, there was no evidence for a significant difference in the proportions of overlapping genotype calls across replicates at all sites (*p* = 0.89) nor at high-confidence sites (GQ > 20, *p* = 0.80) when comparing data from low- and high-pass kits, suggesting distributions of genomic coverage are similar (Fig. S1d). At 1x coverage, the proportion of all sites overlapping was substantially lower than at 4x (medians of 0.101 and 0.435, respectively), demonstrating the random nature of genomic coverage across low-pass replicates. When examining genotype call concordance at 4x coverage, we again found no evidence for a significant difference at all sites (*p* = 0.10) nor at high-confidence sites (*p* = 0.068) between the low- and high-pass kit (Fig. S1e). At 1x coverage, we observed a reduction in genotype concordance at high-confidence sites when compared to 4x coverage (medians of 0.931 and 0.978 respectively), which is likely driven by lower overall genotype qualities in the former (Fig. S1c).

Finally, we evaluated variant calls across the library kits for 4 of the 12 individuals that are also part of the 1000 Genomes high-coverage call set [32]. Using the high coverage call set as ground truth and measuring recall, precision, and non-reference concordance rates (NCR) (see Methods, Fig. S1f-h) we observed no significant difference between kits in recall (*p* = 0.97 and *p* = 0.61 for low- and high-confidence sites, respectively, Wilcoxon rank sum test) or NCR (*p* = 0.74 and *p* = 0.12) and a small but significant 0.93% increase in precision with the high-pass kit at low-confidence sites (*p* = 0.0027; *p* = 0.30 for high-confidence sites). Furthermore, we observed that while NCR improves with GQ (Fig. S1h), there is a large fraction of true variants at low GQ (Fig. S1f) that would get filtered out with standard thresholds such as GQ20.

Based on our observations, we conclude that low-pass optimized kits are suitable for mid-pass applications at a target coverage range of approximately 4x.

### Optimized joint variant calling and imputation by combining high- and mid-pass whole genome sequencing

Having established a cost-effective and scalable method for generating sequencing libraries for mid-pass applications, we next sought to apply the strategy to genotyping an ethnically-diverse cohort lacking an existing reference panel for imputation. To date, individuals of Polynesian ancestry have been underrepresented in genomic studies and are not present in commonly used multi-ancestral reference panels such as 1000 Genomes [33]. To this end, we performed a combination of high- and mid-pass WGS on a cohort of 1510 individuals of Polynesian ancestry recruited from the Māori and Pacific populations of Aotearoa New Zealand (Fig. S2). We sequenced the genomes of 100 individuals at a median coverage of 35.2x using the high-pass WGS library kit and all 1510 at a median coverage of 3.67x using the low-pass library kit (see Methods). A subset of the cohort had previously had their genomes sequenced using high-pass WGS (*n* = 106 individuals, median coverage 37.9x) and genotyped using an array (*n* = 1293 individuals) [34]. Together, these provide an optimal data set for methods development and benchmarking. Throughout the following analyses, we used the pre-existing 30x high-pass WGS data as a truth set to assess recall, precision, and non-reference concordance rates (NCR) of genotype calls derived from the mid-pass approach (see Methods). Unless otherwise noted, we restricted analyses to high-confidence regions of the genome which exclude difficult-to-map regions such as segmental duplications and other highly repetitive sequences as have been defined by the Genome In a Bottle Consortium [35].

**Fig. 1:**
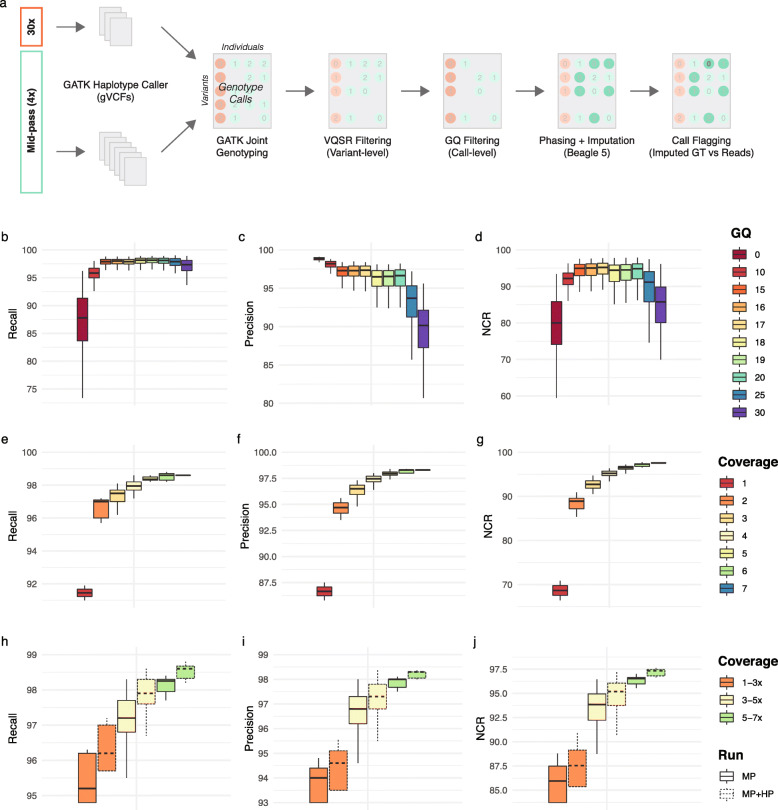
Optimized methods for genotyping from mid-pass whole genome sequencing. **a**) Outline of strategy for variant calling and imputation using a combination of 100 high- and 1410 mid-pass sequenced genomes. Recall, precision, and non-reference concordance rate (NCR) calculated for imputed genotypes derived from mid-pass sequencing of the genomes of 92 individuals as a function of: pre-imputation call-level filtering using the GQ metric (keeping variants with GQ > X, **b-d**), binned sequencing coverage (**e-g**), and both coverage and with (MP + HP, dotted lines) or without (MP, solid lines) inclusion of high-pass sequencing from 100 individuals in joint-calling and imputation (**h-j**). For runs without high-pass included (MP), data for the 100 individuals was substituted with mid-pass data. Metrics were calculated using previously available genotype calls derived from 30x whole-genome sequencing as a truth set. All metrics plotted were calculated for SNVs only (for indels see Figs. S3, S5, and S6). For boxplots, bottom whisker: Q1–1.5*interquartile range (IQR), top whisker: Q3 + 1.5*IQR, box: IQR, center: median, and outliers are not plotted for ease of viewing

We hypothesized that for populations without existing reference panels, inclusion of high-pass data alongside mid-pass data would improve genotype calls. Our strategy to combine mid- and high-pass data was to perform individual-level calling followed by joint genotyping and within-cohort imputation using standard software and best practices (Fig. 1a) [29, 36–38]. While bespoke methods exist for low-pass data [39], we believed that it would be more desirable at mid-pass to optimize a widely established pipeline in order to facilitate broad adoption of the approach (see Methods). Following standard site-level filtering using variant quality score recalibration (VQSR), we performed stringent call-level filtering. We reasoned that low-quality calls, which are abundant at lower coverage levels, would negatively impact the performance of imputation. However, too stringent filtering would result in too few markers for imputation and also negatively impact performance. Thus, we sought to identify an optimum value of genotype call filtering that maximized imputation performance. For imputation, we again deployed a commonly used software with standard settings, so as to maximize compatibility with existing pipelines [30]. By testing a range of GQ filtering values (> 0–30) and comparing post-imputation genotype calls to the 30x truth set, we found that using variant calls with GQ > 17 provided an optimal balance of recall, precision, and NCR for single nucleotide variants (SNV) (Fig. 1b-d). While we primarily focused on SNVs since they make up the vast majority of variant calls, we observed the same threshold to be optimal for indels albeit at a slightly reduced overall performance (Fig. S3).

While GQ filtering of low-confidence calls markedly improved imputation performance, it still results in informative data being discarded. To leverage data at sites with filtered genotype calls we devised an approach to compare imputed genotype calls to GQ-filtered calls (Fig. S4a). We characterized variants as belonging to one of four categories: not imputed, or imputed and identical to the filtered call (IM0); imputed and not inconsistent with the filtered call (IM1); and imputed and inconsistent when the filtered call was either heterozygous (IM2) or homozygous (IM3). “Inconsistent” here is defined as the loss or disappearance of an allele after imputation. Given the nature of mid-pass sequencing data, where we expect to frequently only observe one of two alleles due to low coverage, we categorize the addition or appearance of an additional allele after imputation as “not inconsistent”. By comparing recall, precision, and NCR across variants stratified by IM flag, we found that, as expected, variants where the imputed genotype was inconsistent with the filtered call had significantly worse performance (Fig. S4d-i). In particular, performance at IM3 variants, where the filtered call was homozygous for one allele and the imputed genotype was homozygous for the other allele, was particularly poor. However, these variants only account for a small fraction of the total number of calls (Fig. S4b-c). Thus, due to the lower NCR, we suggest that calls flagged as IM3 and potentially IM2 be filtered out for any downstream applications that are particularly sensitive to incorrect genotype calls.

Next, using the established GQ filtering threshold, we examined post-imputation performance across coverage levels (Fig. 1e-g, Fig. S5). We observed a steep drop off in overall performance for individuals sequenced at 1x when compared to 2x or greater. At 4x coverage, our optimized best-practices based approach yielded high recall (98.0%), precision (97.5%), and NCR (95.2%). As expected, indel calling performed slightly worse overall, with median recall, precision, and NCR of 96.9, 91.9, and 92.1%, respectively. Extending the analyses to the whole genome (i.e., including repetitive and highly variable regions) still yielded high recall (96.1%) for SNVs at 4x but came with a loss in precision (90.8%) and NCR (92.5%) (Fig. S5a). This loss in precision and NCR was more strongly pronounced for indels (Fig. S5c). Strict filtering criteria would be required to make these variants suitable for downstream analyses.

Finally, our study design allowed us to compare the effectiveness of including high-pass data in the joint genotyping and imputation stages to approaches relying on mid-pass sequencing of the entire cohort. To this end, we produced two imputed call sets and assessed their respective performances: the first included high-pass data for 100 individuals and mid-pass data from 1410 individuals (MP + HP); the second included mid-pass data for all 1510 individuals (MP). Inclusion of high-pass data improved performance across all metrics and coverage levels, but improvements were greater for individuals with low (1-3x) coverage levels for SNVs (recall = + 1.05%, precision = + 0.64%, NCR = + 1.85%, Fig. 1h-j) with more pronounced improvements for indels (recall = + 2.47%, precision = + 2.08%, NCR = + 3.24%). Analyzing performance pre- and post-imputation revealed that the inclusion of high-pass data yielded minor improvements in joint-genotyping precision, but that most improvements came post-imputation (Fig. S6). This suggests that having a subset of high-confidence, complete genotype calls is able to improve imputation in the mid-pass sequenced individuals, albeit to a minor extent.

Based on our analyses, we found that with optimization, standard best-practices based variant calling and imputation pipelines are suitable for genotyping using mid-pass WGS and generate comprehensive and accurate genotype calls. Furthermore, the inclusion of a subset of individuals sequenced at high-pass yielded better performance in the entire cohort, largely through improved imputation performance.

### Mid-pass whole genome sequencing outperforms array-based genotyping for diverse ethnicities

After establishing genotyping methods for mid-pass WGS, we next applied the approach to characterize genetic variation. The cohort comprised individuals with self-reported ethnicities drawn from the Māori and Pacific populations of Aotearoa New Zealand with representation of both Eastern and Western Polynesian nationalities (Fig. S2h).

**Fig. 2:**
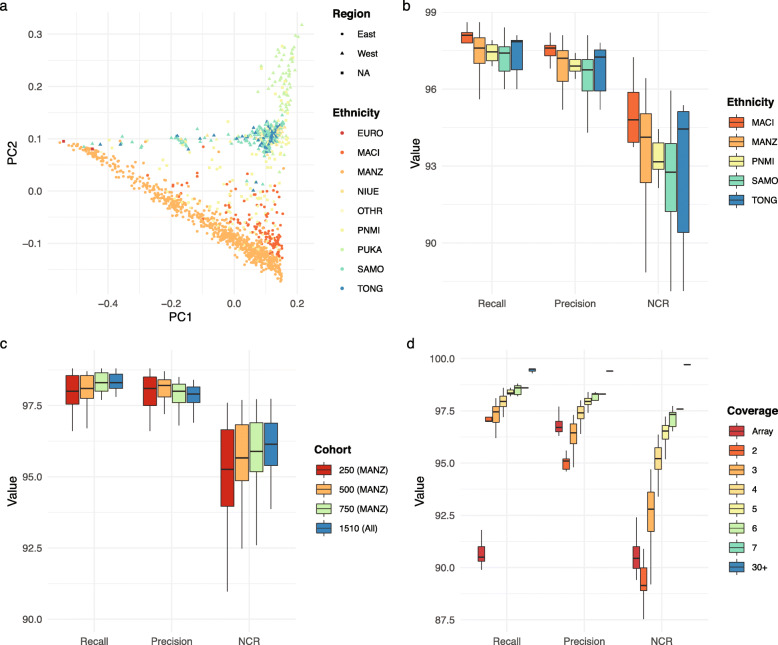
Performance of mid-pass whole genome sequencing across self-reported ethnicities and compared to array genotyping. **A**) Principal component analysis of imputed genotype data from 1410 mid-pass and 100 high-pass sequenced Polynesian individuals’ genomes. Data points are colored by self-reported ethnicity (EURO, European, MACI, Cook Islands Māori, MANZ, Aotearoa New Zealand Māori, NIUE, Niuean, OTHR, other, PNMI, Mixed Ethnicity Polynesian, PUKA, Pukapukan, SAMO, Samoan, TONG, Tongan, listed in alphabetical order) with symbols corresponding to the broader regional division of Polynesia (East, West or NA, not applicable). **B**) Performance measured using recall, precision, and non-reference concordance rate (NCR) for mid-pass derived imputed genotype calls across self-reported ethnicities. Metrics were calculated for the genomes of 100 individuals sequenced as part of this study at both and high- and mid-pass using the high-pass genotype calls as a truth set. **C**) Performance as a function of cohort size for individuals with self-reported Aotearoa New Zealand Māori ethnicity. Individuals were selected such that the smaller cohorts have less European ancestry admixture (Fig. S8). **D**) Performance calculated from imputed genotypes for 84 individuals binned by sequencing coverage with corresponding array data for comparison and using previously available 30x whole-genome sequencing genotype calls as a truth set. For boxplots, bottom whisker: Q1–1.5*interquartile range (IQR), top whisker: Q3 + 1.5*IQR, box: IQR, center: median, and outliers are not plotted for ease of viewing

First we examined principal components (PCs) derived from imputed genotype calls. We found that PC1 was highly correlated with the degree of European ancestry admixture (Spearman’s *ρ* = − 0.89, *p* < 2.2e-16, Figs. 2a, S7). PC2 robustly captured Eastern vs Western Polynesian ancestry, with Samoan and Tongan people clustering at one end of the spectrum, Aotearoa New Zealand Māori clustering at the other, and Cook Island Māori in the middle. Reassuringly, PCs 3 and 4 also clearly corresponded to self-reported Pukapukan and Niuean ethnicities, respectively (Fig. S8). When examining the correlation between PCs and technical factors, we found that PC5 was linearly correlated with log(sequencing coverage) (Fig. S8j).

Next we assessed the performance of our mid-pass approach across self-reported ethnicities. Overall, we found that differences in performance across ethnicities were minor, being smaller in magnitude than differences due to coverage level (Fig. 2b). Performance was best for individuals of self-reported Aotearoa New Zealand and Cook Island Māori ethnicity, most likely because this is the best represented group in the cohort (*N* = 834 combined). Individuals of Western Polynesian nationality (*N* = 298 combined between Samoan and Tongan people) had slightly lower NCR, but comparable recall and precision values to other self-reported ethnicities.

While the full cohort was ethnically diverse, we sought to benchmark our mid-pass approach in the context of smaller, more homogenous cohorts. To do this, we focused on individuals with self-reported Aotearoa New Zealand Māori ethnicity, as this comprised the largest group, and subsetted to 750, 500, and 250 individuals. Subsetting was performed by selecting individuals that showed less European ancestry admixture (Fig. S9a) and the proportion of individuals sequenced at high vs mid-pass was kept approximately constant across the subsets (6.5–9.2%, Fig. S9b). We found that recall, precision, and NCR were similar across the cohort subsets as compared to performance when the entire cohort was used (Figs. 2c, S9c-h). In fact, precision tended to be slightly higher in the smaller, less admixed cohorts, although the differences were minor and the small improvements in recall and NCR slightly more pronounced.

Finally, we compared genotype calls derived from our mid-pass approach to those derived from array-based genotyping followed by imputation into 1000 Genomes, which is commonly used for diverse genomic studies (see Methods). Examining overall recall, precision, and NCR for individuals with truth sets derived from 30x WGS, we found that array genotyping followed by imputation resulted in much lower recall compared to all mid-pass coverage levels (Fig. 2d). Precision however, was comparable to mid-pass individuals sequenced at 3x coverage, while NCR was comparable to mid-pass at 2x coverage. When examining performance as a function of minor allele frequency (MAF) we found that with the exception of precision at low frequency (MAF ≤ 2%) variants, 4x mid-pass WGS outperformed array genotyping with 1000 Genomes imputation across all metrics and frequencies (Fig. S10).

Through these analyses we found that our mid-pass sequencing strategy effectively identified the genetic variation present in a diverse study cohort, was minimally affected by technical covariates, and performed consistently across ethnicities and cohort sizes. When compared to genotyping arrays followed by imputation into 1000 Genomes, mid-pass better identified genetic variation while at the same time having higher recall and precision across most coverage levels.

### Mid-pass sequencing identifies novel, potentially population-specific genetic variation with putative functional impact that is missed by imputation into 1000 Genomes

**Fig. 3:**
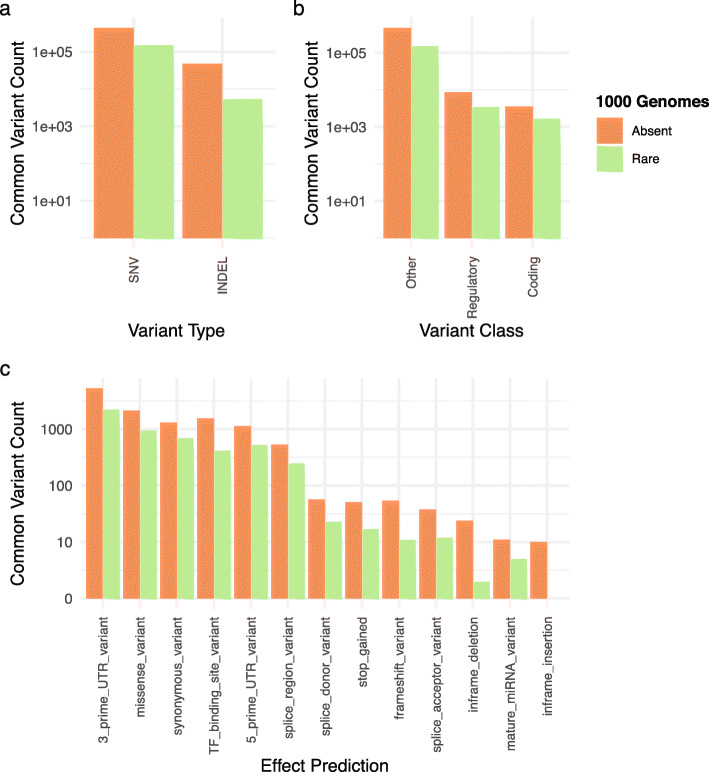
Functional annotation of putatively Polynesian enriched variants identified by mid-pass sequencing. Variants are characterized as being absent (orange) or rare (MAF < 1%, green) in 1000 Genomes Phase 3 and common (MAF > 5%) in the study dataset. Breakdown of variants as a function of type (SNV/INDEL, **A**), class (coding, regulatory, or other, **B**), and predicted effect (**C**). Indels located in high-confidence regions of the genome and all SNVs were included in the analysis. Variant counts (y-axis) have been log-transformed for ease of viewing

A major advantage to whole genome sequencing as compared to array genotyping is the ability to detect novel genetic variation that could contribute to disease etiology. For our final analyses, we examined genetic variation that is detected using our approach but missed when using array genotyping followed by imputation into 1000 Genomes. We characterized variants that were common (MAF > 5%) in the study cohort and either absent from or rare (MAF < 1%) in 1000 Genomes (Fig. S11). Mindful that this may enrich for false positive genotype calls, we restricted our analyses to all SNVs, where our analyses showed false-positive rates to be low (Fig. S5a), and only included indels found in high-confidence regions of the genome (Fig. S5d). Using this approach, we identified 426,256 SNVs and 44,235 indels that were common in the cohort but absent from 1000 Genomes, and an additional 152,113 SNVs and 5475 indels were rare in 1000 Genomes (Fig. 3a). Further classifying these variants based on predicted class revealed 22,649 regulatory and 3514 coding variants that were absent from 1000 Genomes, representing a substantial amount of potentially Polynesian-specific genetic variation with putative functional impact (Fig. 3b). Finally, we examined the predicted effect of genetic variation, as this is often of primary importance in genomic studies (Fig. 3c). Mid-pass sequencing identified 155 putative loss of function variants (across splice donor, splice acceptor, stop-gained, and frameshift variants) as well as 2089 missense variants that were absent from 1000 Genomes, but common in the study cohort. When examining variants with putative regulatory impact we found 14,264 regulatory region variants, 6247 UTR, 1521 transcription factor binding site, and 519 splice-region variants that were absent from 1000 Genomes and common in the study cohort.

## Discussion

From our analyses of kits for library generation, we conclude that those optimized for low-pass sequencing facilitate high-throughput processing while providing data that are largely consistent with high-pass kits. The pooled processing of a high number of samples offered by low-pass kits reduces variability of coverage, number of batches, and cost, which is especially important for low-pass studies. However, a notable drawback is that we observed higher duplication levels when a low-pass kit was used. This could be a result of the much smaller amount of input DNA used for the low-pass kit (5–25 ng) vs the high-pass kit (200 ng), resulting in lower library diversity. Based on this observation, we would not suggest exceeding target coverage levels of 4x using current generation low-pass optimized library preparation methods because of diminishing returns attributed to increased read duplication rates. However, at a target coverage level of 4x, we believe the workflow and cost benefits of low-pass optimized kits outweigh the drawbacks from increased duplication rates. If higher coverages are desired, we suggest using high-pass library preparation kits. We have made the sequencing data from HapMap individuals generated for library kit benchmarking publicly available to facilitate broader adoption of low- and mid-pass sequencing strategies and to further encourage development of computational genomic methods (see Data and Code Availability).

In this work we sought to develop and share a bioinformatics pipeline for processing mid-pass data that made use of commonly used software packages. We reasoned that such a pipeline would be more accessible, as many users would already be familiar with the various components. However, we found that our “off-the-shelf” approach fared poorly at coverage levels ≤1x. It is very likely that at this coverage level, low-pass optimized variant calling and imputation methods would perform much better than our more standard approach [39].

With respect to downstream analyses that make use of mid-pass data, such as GWAS, we suggest filtering out imputed calls that are discordant with sequencing reads and provide scripts to do so (see Data and Code Availability). Furthermore, while the mid-pass approach yielded high-quality genotype data that largely correlated with self-reported ethnicity, we found that coverage level did introduce sources of variation. Thus, we suggest that any downstream applications should appropriately control for this technical factor.

We also explored the performance of various mid-pass study designs. We found that sequencing the genomes of a subset of individuals at high-pass and including them in joint-genotyping and imputation led to minor improvements in performance across the entire cohort. While these improvements were small, there are other potential benefits to including high-pass sequenced genomes that are not explored here. For example, variants that are harder to identify from short-read data such as large indels and structural variants may benefit more substantially from high-pass data and could then be genotyped in individuals with mid-pass data. We also explored the performance of the mid-pass approach as a function of study size. For more homogenous cohorts, we found that mid-pass WGS performed well even at a cohort size of just 250 individuals, making it a robust approach for smaller scale studies.

It is important to consider the strengths and weaknesses of mid-pass WGS in the broader context of genotyping strategies. At present, researchers carrying out population-scale genomic studies have options including 30x WGS, whole exome sequencing (WES), mid−/low-pass WGS, and genotyping arrays, roughly in order of cost. Ultimately, there is not one perfect solution, so researchers must decide on the strategy that best fits their needs and budget. Based on our work, we would suggest mid-pass WGS for cost-effective, population-scale genetic studies of individuals that are not well represented in existing genomic reference panels. This is a particularly effective approach for studies that aim to identify variants that are rare globally but common locally [40]. For population-scale genetic studies of European ancestry individuals, low-pass WGS has been demonstrated to be a highly effective strategy [15]. When high confidence, individual-level genotypes for rare protein-coding variants with predictable effects are of the utmost importance, for example in clinical studies, WES remains the most effective strategy, despite its narrow scope and relatively high cost. However, the lack of genome-wide data from WES makes it much less powerful for population-scale studies, and usually necessitates the generation of additional complementary data from genotyping arrays.

In the context of diverse genomic studies, a recent cost-effective approach has been to use WGS to sequence the genomes of a subset of individuals in the population of interest, and use these data to design custom arrays that capture population-specific variants and/or produce a population-specific reference panel for imputation use. While this has been an effective interim solution, the power to detect novel genetic variation is still limited to those few individuals selected for WGS, unlike mid-pass approaches where novel variant discovery is possible in every individual. In addition, the process of designing custom arrays is in itself costly and time intensive. Thus, we believe the advantages of mid-pass WGS outweigh this approach.

Finally, we present mid-pass WGS not just as a cost-saving strategy for generating genomic datasets with populations that have previously been underrepresented in human genetic research, but also as a means to democratize statistical and population genetic tools. For over a decade, GWAS and polygenic score methodologies have been applied, optimized, and reapplied to cohorts of largely Western European ancestry. Consequently, public health outcomes from genomics research disproportionately benefit individuals of Western European ancestry and globally reinforce institutional disparities that Indigenous communities are actively fighting to dismantle [41, 42].

Our mid-pass sequencing approach identified many potentially population-specific variants with functional impact potentially important in disease etiology that would have been missed by array genotyping followed by imputation into 1000 Genomes. Common population-specific variants are implicated in metabolic disease in Polynesian populations. The Gln allele of the CREBRF p.Arg457Gln variant associates with increased BMI but reduced risk of diabetes [34], the Ser allele of the IL37 p.Asn182Ser variant with gout [43], and the Western Polynesian-specific Leu allele of the ABCC4 p.Pro1036Leu variant with gout [44]. The Māori and Pacific populations of Aotearoa New Zealand are affected by a range of polygenic conditions such as type 2 diabetes, gout, and other diseases with a metabolic basis. The underpinning genetic causes differ to various extents relative to the larger European population [45]. While structural inequities contribute to the increased prevalence [43, 46], we expect population-specific genetic variants to contribute to health status. Studying these population-specific variants will provide insights into disease pathogenesis directly relevant to Māori and Pacific people.

While mid-pass WGS provides a technical solution to overcome the lack of diversity in genomics research, greater change in research practices will be needed to course-correct human genetics. Specifically, given that Western researchers have a documented history of ignoring, overlooking, and abusing BIPOC (Black, Indigenous, people of color) populations, sweeping revisions of how underrepresented peoples are engaged in genomics research are required [4, 11]. Rather than imposing Western perspectives of genetic privacy, data sharing, and disease priorities, researchers should engage in community-led partnerships that empower participants of genetic research to define the parameters under which their genomes are studied [47, 48]. Lastly, partnerships should bring impactful changes not only to science and medicine but also to participants and their communities. Approaches that ethically engage populations, build genomics capacity, and return both short and long-term benefits are long overdue.

## Conclusion

Our work has demonstrated that mid-pass WGS is a cost-effective strategy for generating high quality genomic datasets from diverse populations without reliance on external datasets or reference panels. In order to maximize adoptability, we have established a framework for mid-pass WGS that uses commercially available reagents and optimized pipelines consisting of widely used software packages. Methods and approaches that improve accessibility and affordability will empower researchers around the world to carry out their own genomic studies and improve global diversity in genomic studies.

## Methods

### HapMap sequencing pilot

We selected 12 HapMap individuals (NA12877, NA12878, NA12879, NA18954, NA18995, NA19238, NA24143, NA24149, NA24385, NA24631, NA24694, NA24695) to test sequencing coverage and data quality of low-pass kits. We generated 10 replicates per DNA sample: 4 replicates at 1x using plexWell LPS384 library preparation, targeting 150Gb total bases (corresponding to 3.125Gb per sample on average); 4 replicates at 4x using plexWell LPS384 library preparation, targeting 600Gb total (12.5Gb per sample); 2 replicates at 4x using plexWell WGS24 library preparation, targeting 300Gb total (12.5Gb per sample). Libraries were sequenced on NovaSeq 6000 instruments, with 2x151bp reads. Library preparation and sequencing were done at Psomagen Inc. (USA).

### Aotearoa New Zealand study participants

Individuals of self-reported Māori and / or Pacific ethnicity aged ≥16 years, primarily from the Auckland, Waikato, and Christchurch regions of Aotearoa New Zealand, were recruited to the Genetics of Gout, Diabetes, and Kidney Disease in Aotearoa New Zealand Study. 65 participants were recruited as part of a partnership with the Pukapuka Community Group (Mangere, South Auckland). The cohort consisted of 716 males and 794 females and the median age was 47 years. 421 individuals were diagnosed with kidney disease, 247 were diagnosed with gout, and 438 were diagnosed with type 2 diabetes. 908 individuals had not been diagnosed with any of the above.

### DNA sequencing of 1510 mid-pass and 100 high-pass whole genomes

DNA was extracted in Aotearoa New Zealand and shipped to Psomagen Inc. (USA) for library preparation and sequencing. Mid-pass libraries were prepared using plexWell LP384 kits and subsequently sequenced targeting an average of 16Gb per sample. We targeted a higher coverage than for the HapMap sequencing pilot to account for the higher duplication rate observed when using the plexWell kits. In addition, when pooling large numbers of samples some variation in coverage is unavoidable, so targeting a higher coverage ensures more samples will be in a usable coverage range for our purposes (>1x). High-pass libraries were prepared using TruSeq PCR-Free (350 bp) kits and sequenced at a target coverage of 30x per sample. All sequencing was done on NovaSeq 6000 instruments with 2x151bp reads. After sequencing and QC, DNA samples were returned to Aotearoa New Zealand to be disposed of in a culturally appropriate manner.

### Pre-existing whole genome and array data

Previous to this study, 106 individuals from the cohort had their genomes sequenced to high coverage (TruSeq Nano libraries sequenced on HiSeqX) and 1293 individuals were genotyped using Illumina Infinium CoreExome arrays (v1.0–1.3) [34]. We reprocessed the 106 whole genomes to obtain joint variant calls as detailed below. For the array data, GRCh37 genotype calls were lifted over to GRCh38, resulting in 471,499 genotyped positions on autosomes which were then further imputed into the 1000 Genomes mapped onto GRCh38 reference panel [49] using Beagle v5.1 [30] (beagle.27Apr20.b81.jar).

### Processing of whole genome data

Raw sequencing data were inspected with fastqc (v0.11.7) and adapters were trimmed using cutadapt (v2.10). Trimmed reads were then processed following the GATK Best Practices guidelines [29] (BWA-mem v.0.7.15, GATK v.4.1.4.0) to produce joint-called and VQSR-filtered multi-sample VCFs. We set --truth-sensitivity-filter-level to 99.8 for SNPs and 99.0 for indels in VQSR and only retained PASS filter sites for further analyses. The GATK Best Practices guidelines are thoroughly outlined here: https://gatk.broadinstitute.org/hc/en-us/articles/360035535932-Germline-short-variant-discovery-SNPs-Indels-, and we provide some key command lines as well in https://github.com/variant-bio/mid-pass. For single sample genotyping we ran GenotypeGVCFs on HaplotypeCaller-generated individual GVCF files without any additional filtering (unless GQ thresholds where indicated).

### GQ filtering, imputation, and site flagging

We used custom scripts to filter genotype calls below our chosen threshold of GQ < =17. Subsequently, Beagle v5.1 [30] (beagle.27Apr20.b81.jar) was run without a reference panel and specifying the ‘gt=’ input parameter for within-cohort imputation. The resulting VCFs were re-merged with the original unfiltered VCF to flag sites by consistency with the filtered calls. Call flagging script as well as command lines used can be found at https://github.com/variant-bio/mid-pass.

### Performance evaluation data

The genomes of 106 individuals with previously available 30x WGS data were joint-genotyped as detailed above and all PASS filter variants and genotypes with GQ > 20 were used as truth set in the subsequent evaluations. For MAF-based analyses, we additionally applied a variant call rate filter of 50%. Out of the 93 individuals with mid-pass and 30x reference data available (Fig. S2), one individual was excluded from evaluations due to sample contamination, resulting in 92 genomes total for most of the evaluations. Comparisons including array data were limited to the genomes of 84 individuals for which array data were additionally available. Cohort size evaluations were limited to include individuals with self-reported Aotearoa New Zealand Māori ethnicity, and individuals were included based on PC1 and PC2 to form sub-cohorts of 250, 500, and 750 individuals (Fig. S8a). Cohort size evaluations were limited to the genomes of 22 individuals that were part of the smallest sub-cohort and for which both MP and 30x reference data were available.

### Performance assessment methods

Throughout the performance assessments, we used recall, precision, and non-reference concordance (NCR) to assess accuracy of variant calls. Recall and precision serve as (coordinate-based) variant site metrics whereas NCR further assesses allele and genotype accuracy. Recall is defined as the number of true positive variant sites divided by the total number of variant sites in the truth set. Precision is defined as the number of true positive variant sites divided by the total number of variant sites in the test set. Non-reference concordance is the fraction of correctly called genotypes, excluding homozygous reference matches. For MAF-based performance comparisons, we used minor allele concordance rather than non-reference concordance, i.e. alt and ref. allele were flipped where variant allele frequency was greater than 0.5. High-confidence regions of the genome were defined as regions present in the “GRCh38_notinalldifficultregions.bed “file provided by the Genome in a Bottle Consortium [35] and described in https://opendata.nist.gov/pdrsrv/mds2-2190/GRCh38/union/v2.0-GRCh38-Union-README.txt. Pipeline optimization (Fig. 1) as well as subcohort experiments (Fig. 2c) were limited to chromosome 1 only. Comparisons on imputed array data were limited to autosomes. All other comparisons were genome-wide excluding chrY and alt contigs. All pairwise comparisons between test and reference genotype call sets were done using vcf-compare (v0.1.14–12-gcdb80b8).

### Principal component analysis

PCA was performed on imputed genotypes using Hail v0.2. Briefly, for individuals with ≥1.5x mean coverage, SNPs and indels in high-confidence regions with MAF > 1% and imputation rate < 30% were LD-pruned using the ld_prune() function with parameters r2 = 0.2, bp_window = 100,000. Principal components were calculated using LD-pruned variants using the hwe_normalized_pca() function with parameters, k = 20.

### European admixture analysis

Genotype calls for 91 British individuals (GBR) from 1000 Genomes high coverage sequencing and mapping to GRCh38 were downloaded [32]. Chromosome 1 was subsetted, and merged with the genotype calls from Polynesian individuals using the Hail function union_cols(), which performs an inner join on the two call sets. Allele frequencies were calculated in the combined call set and variants were filtered based on MAF > 1%. To remove indels and multiallelic variants*,* VCF files were subset to biallelic SNPs using *bcftools.* Thereafter, to thin variation, LD pruning was performed in PLINK (v1.90b6.16) with settings *--indep-pairwise 50 10 0.1* followed by random down-sampling of the remaining variation with settings *--thin-count 245,000*. Using the resulting set of 245,000 biallelic SNPs as input for ADMIXTURE (v1.3.0), we estimated ancestry-specific allele frequencies and fractions assuming k = 2.

### Overlap with 1000 genomes variants and functional annotation

All variants with MAF > 5% in the study cohort, excluding indels in non-high-confidence regions, were compared to 1000 Genomes phase 3 variants. Variants were classified as either rare (< 1% MAF in 1000 Genomes) or novel (absent from 1000 Genomes) and annotated for functional impact using Variant Effect Predictor with cache version 102 (homo_sapiens_vep_102_GRCh38). Variant class was defined as follows: coding (frameshift_variant, inframe_deletion, inframe_insertion, missense_variant, start_lost, stop_gained, stop_lost, stop_retained_variant, synonymous_variant), regulatory (5_prime_UTR_variant, 3_prime_UTR_variant, mature_miRNA_variant, regulatory_region_variant, splice_acceptor_variant, splice_donor_variant, splice_region_variant, TF_binding_site_variant, TFBS_ablation), and other (downstream_gene_variant, intergenic_variant, intron_variant, non_coding_transcript_exon_variant, upstream_gene_variant).

## Supplementary Information

**Additional file 1: Figure S1.** Benchmarking of libraries generated with low-pass kits sequenced at intermediate coverage levels. a) Mean coverage across the library types. b) Per-sample duplicate rate over (deduplicated) sequencing coverage. c) Genotype quality (GQ) as a function of mean GQ (averaged over 2 × 12 samples). Fraction of variant calls that overlap between replicates (d), and their genotype concordance (e) at either all variants (GQ > 0) or high-confidence variants (GQ > 20). f) Recall, g) Precision and h) Non-reference concordance rates computed per sample against the 1000 Genomes high coverage call set [32] as “truth”. The single HP4 sample with coverage>10x was excluded from this comparison. **Figure S2.** a) Overview of data types available for participants and how they overlap. b) Distribution of de-duplicated sequencing coverage per sample for low-pass samples, c) TruSeq PCR-free high-coverage samples, d) TruSeq Nano high-coverage samples. e) Distribution of sequencing duplicate rates per sample for low-pass samples, f) for TruSeq PCR-free samples, and g) for TruSeq Nano samples. h) Breakdown of number of individuals by self-reported ethnicity and sequencing type. **Figure S3.** Effect of GQ filtering on indel calling performance. a) Recall, b) Precision, and c) NCR for indels over varying GQ thresholds. **Figure S4.** Accuracy of flagged sites by flag type. a) Overview of the different flag types that characterize variants by comparing (filtered) sequencing-based genotype with genotype after imputation. A call is flagged with IM = 0 if sequencing-based genotype and imputed genotype agree fully. Given low coverage, we consider the lack of sequencing data evidence for an imputed allele as “not inconsistent” while the disappearance of an allele after imputation is categorized as “inconsistent”. IM = 1 therefore flags imputed calls that are not inconsistent with the sequencing-based call (either because it was missing or we may have only observed one of two alleles in sequencing). IM = 2 and IM = 3 flag sites that are inconsistent between sequencing-based and imputed calls, where IM = 2 calls were heterozygous in sequencing (potentially due to sequencing or mapping artifacts or contamination, or an error in imputation) and IM = 3 calls were homozygous for the opposite allele. b) Fraction of SNV calls in each IM flag category. c) Fraction of indel calls in each IM flag category. d) Recall (normalized to each individual’s overall SNV recall), e) Precision, and f) NCR of SNVs. g) Normalized recall, h) Precision, and i) NCR of indels. **Figure S5.** Detailed performance (recall, precision, and NCR) of SNV and indel calling both genomewide (including repetitive regions) as well as in high-confidence regions only, shown over coverage. a) SNVs genomewide, b) SNVs in high-confidence regions, c) Indels genomewide, d) Indels in high-confidence regions. **Figure S6.** Performance comparison across different pipeline stages/runs. a) Overview of tested call sets. “Single” refers to individually called mid-pass data (GQ > 17). “MP” and “MP-HP” refer to the joint-called (“joint”) and imputed (“imp”) call sets using mid-pass data from 1510 individuals (MP) and mid-pass data from 1410 individuals plus high-pass data from 100 high-pass individuals (MP-HP) For more details see methods. b) Recall, c) Precision, and d) NCR for SNVs. e) Recall, f) Precision, and g) NCR for indels. **Figure S7.** Analysis of European admixture in the study cohort. ADMIXTURE was run assuming two populations on the cohort with 91 British individuals from 1000 Genomes (GBR) included to capture European ancestry. Shown are the proportions of ancestry estimated (population 1 = red, population 2 = orange). Individuals are ordered by cohort (GBR/Polynesian). Analysis of PC1 from PC analysis versus proportion of population 1 ancestry from ADMIXTURE analysis found that PC1 is highly correlated with the degree of estimated European ancestry (Spearman’s *ρ* = − 0.89, *p* < 2.2e-16). **Figure S8.** Principal component (PC) analysis of imputed genotype calls. a-i) PC1 vs PC2–10, labeled based on self-reported ethnicity. j) PC5 vs log(coverage) with data points colored by sequencing depth and symbols corresponding to library type (plexWell LP384 used for low-pass sequencing and TrueSeq PCR-Free used for high-pass sequencing). Individuals with ≥1.5x coverage and both SNVs and indels in high-confidence regions of the genome were used for the analyses. **Figure S9.** Effect of cohort size on performance. a) PCA of self-reported Aotearoa New Zealand Māori individuals that were included in the analysis. b) Sequencing type breakdown within subcohorts (MP, mid-pass, HP, high-pass). c) Recall, d) Precision, and e) NCR for SNVs. f) Recall, g) Precision, and h) NCR for indels. **Figure S10.** MAF-based comparison of variants in high-confidence regions, split by coverage level. a) Recall, b) Precision, and c) NCR of SNVs over the full MAF range. Panels d), e), and f) show the same plots zoomed in on the 0–7.5% MAF range. Panels g) to l) show the same for indels. **Figure S11.** Allele frequency distribution of common (MAF > 5%) variants in the study cohort that are either absent from (a) or rare in (b) 1000 Genomes. Indels located in high-confidence regions of the genome and all SNVs were included in the analysis.

## Data Availability

Sequencing data from the 120 libraries generated for the 12 HapMap individuals has been deposited to the NCBI BioProject database under accession number PRJNA697982. An outline of the mid-pass variant calling pipeline including tool command lines used and code to flag sites for downstream filtering can be found on Github at https://github.com/variant-bio/mid-pass. Scripts are made available for non-commercial use only. The Polynesian genetic data generated in this study are protected and cannot be made publicly available in respect of the consent and IRB regulations. Data may be available from T.R.M. on a collaborative basis for research consistent with participant consent. The 1000 Genomes datasets are documented and accessible through the 1000 Genomes website: https://www.internationalgenome.org/data-portal/data-collection. Specifically, the unphased high-coverage 1000 Genomes Project data (used for HapMap recall/precision/NCR calculations as well as European admixture analysis) was downloaded from http://ftp.1000genomes.ebi.ac.uk/vol1/ftp/data_collections/1000G_2504_high_coverage/working/20201028_3202_raw_GT_with_annot/, the phased 1000 Genomes data mapped on GRCh38 (used as imputation reference panel for genotyping array data) was downloaded from http://ftp.1000genomes.ebi.ac.uk/vol1/ftp/data_collections/1000_genomes_project/release/20190312_biallelic_SNV_and_INDEL/, and the official Phase 3 data lifted over to GRCh38 (used for allele frequency and functional impact comparisons) was downloaded from http://ftp.ensembl.org/pub/data_files/homo_sapiens/GRCh38/variation_genotype/.

## Abbreviations

BIPOC: Black, Indigenous, people of color
GQ: Genotype quality
HP: High-pass
Indel: Insertion deletion
LP: Low-pass
MAF: Minor allele frequency
MP: Mid-pass
NCR: Non-reference concordance rate
PC: Principal component
SNV: Single nucleotide variant
VQSR: Variant quality score recalibration
WES: Whole exome sequencing
WGS: Whole genome sequencing
