# Supplementary material for: Mid-pass whole genome sequencing enables biomedical genetic studies of diverse populations
Source: BMC Genomics. 2021 Nov 1;22:666. doi: 10.1186/s12864-021-07949-9 (PMC8559369; doi:10.1186/s12864-021-07949-9)
Supplement: Supplementary file 1 — Additional file 1: Figure S1. Benchmarking of libraries generated with low-pass kits sequenced at intermediate coverage levels. a) Mean coverage across the library types. b) Per-sample duplicate rate over (deduplicated) sequencing coverage. c) Genotype quality (GQ) as a function of mean GQ (averaged over 2 × 12 samples). Fraction of variant calls that overlap between replicates (d), and their genotype concordance (e) at either all variants (GQ > 0) or high-confidence variants (GQ > 20). f) Recall, g) Precision and h) Non-reference concordance rates computed per sample against the 1000 Genomes high coverage call set [32] as “truth”. The single HP4 sample with coverage>10x was excluded from this comparison. Figure S2. a) Overview of data types available for participants and how they overlap. b) Distribution of de-duplicated sequencing coverage per sample for low-pass samples, c) TruSeq PCR-free high-coverage samples, d) TruSeq Nano high-coverage samples. e) Distribution of sequencing duplicate rates per sample for low-pass samples, f) for TruSeq PCR-free samples, and g) for TruSeq Nano samples. h) Breakdown of number of individuals by self-reported ethnicity and sequencing type. Figure S3. Effect of GQ filtering on indel calling performance. a) Recall, b) Precision, and c) NCR for indels over varying GQ thresholds. Figure S4. Accuracy of flagged sites by flag type. a) Overview of the different flag types that characterize variants by comparing (filtered) sequencing-based genotype with genotype after imputation. A call is flagged with IM = 0 if sequencing-based genotype and imputed genotype agree fully. Given low coverage, we consider the lack of sequencing data evidence for an imputed allele as “not inconsistent” while the disappearance of an allele after imputation is categorized as “inconsistent”. IM = 1 therefore flags imputed calls that are not inconsistent with the sequencing-based call (either because it was missing or we may have only observed one of two allele [file 12864_2021_7949_MOESM1_ESM.docx]

##

##

## Supplemental Information

## “Mid-pass whole genome sequencing enables biomedical genetic studies of diverse populations”

Contents:

1. Evaluation of low pass library preparation, sequencing, and variant calling
2. Study design and cohort description
3. Pipeline optimization: effect of GQ filtering on indels
4. Pipeline optimization: performance at flagged sites
5. Detailed performance of SNV & indel calling over coverage
6. Effect of joint variant calling and inclusion of high-coverage data
7. European admixture analysis
8. Principal component analysis
9. Effect of cohort size on variant calling and imputation
10. Performance comparison with array data imputed into 1000 Genomes
11. Allele frequency distribution of rare and novel variants

**S1. Evaluation of Low-Pass Library Preparation, Sequencing, and Variant Calling**

**
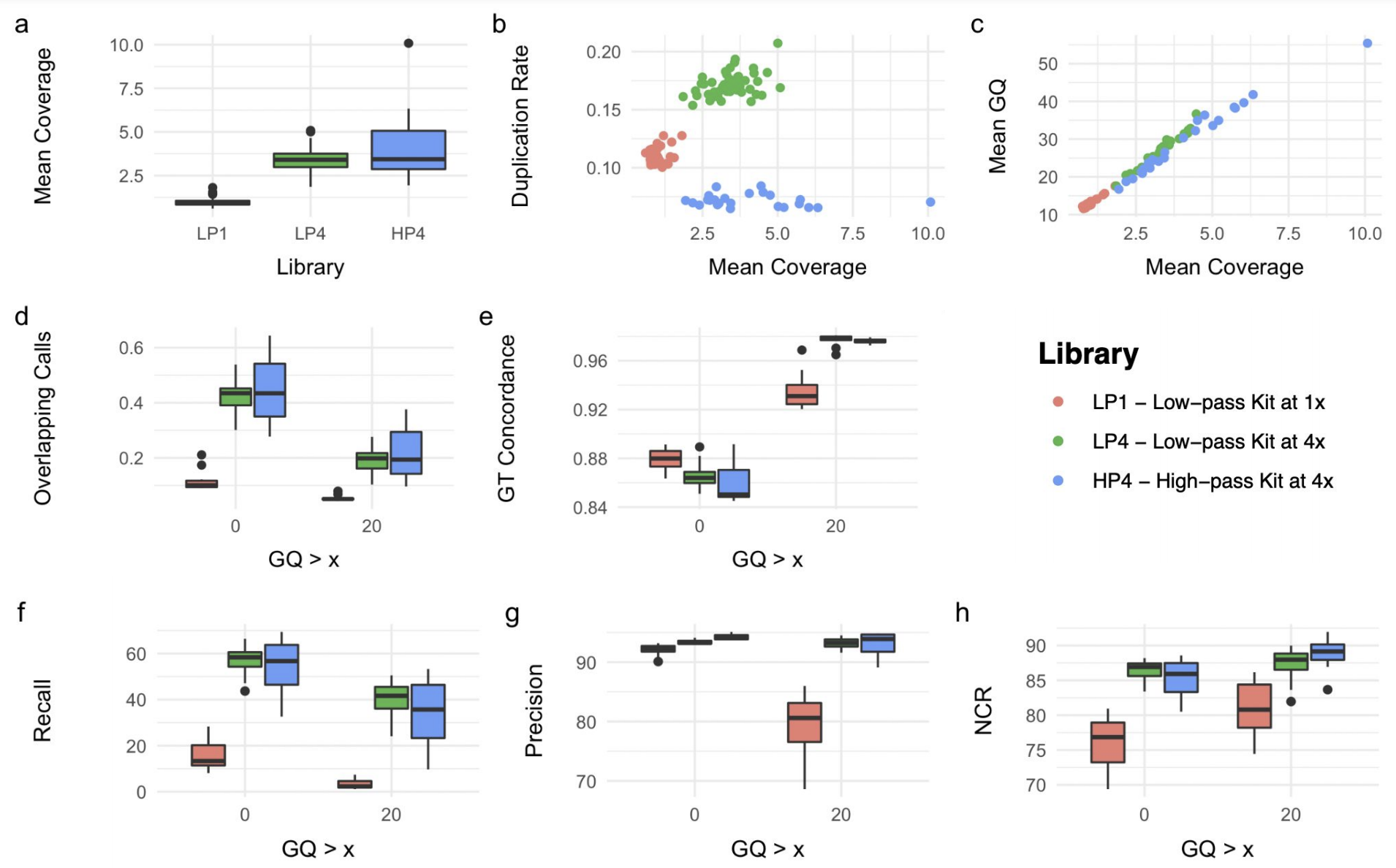
**

**Figure S1.** Benchmarking of libraries generated with low-pass kits sequenced at intermediate coverage levels. a) Mean coverage across the library types. b) Per-sample duplicate rate over (deduplicated) sequencing coverage. c) Genotype quality (GQ) as a function of mean GQ (averaged over 2 x 12 samples). Fraction of variant calls that overlap between replicates (d), and their genotype concordance (e) at either all variants (GQ>0) or high-confidence variants (GQ>20). f) Recall, g) Precision and h) Non-reference concordance rates computed per sample against the 1000 Genomes high coverage call set[^32^](https://paperpile.com/c/bzoM2O/NrTXf) as “truth”. The single HP4 sample with coverage>10x was excluded from this comparison.

**S2. Study Design and Cohort Description**


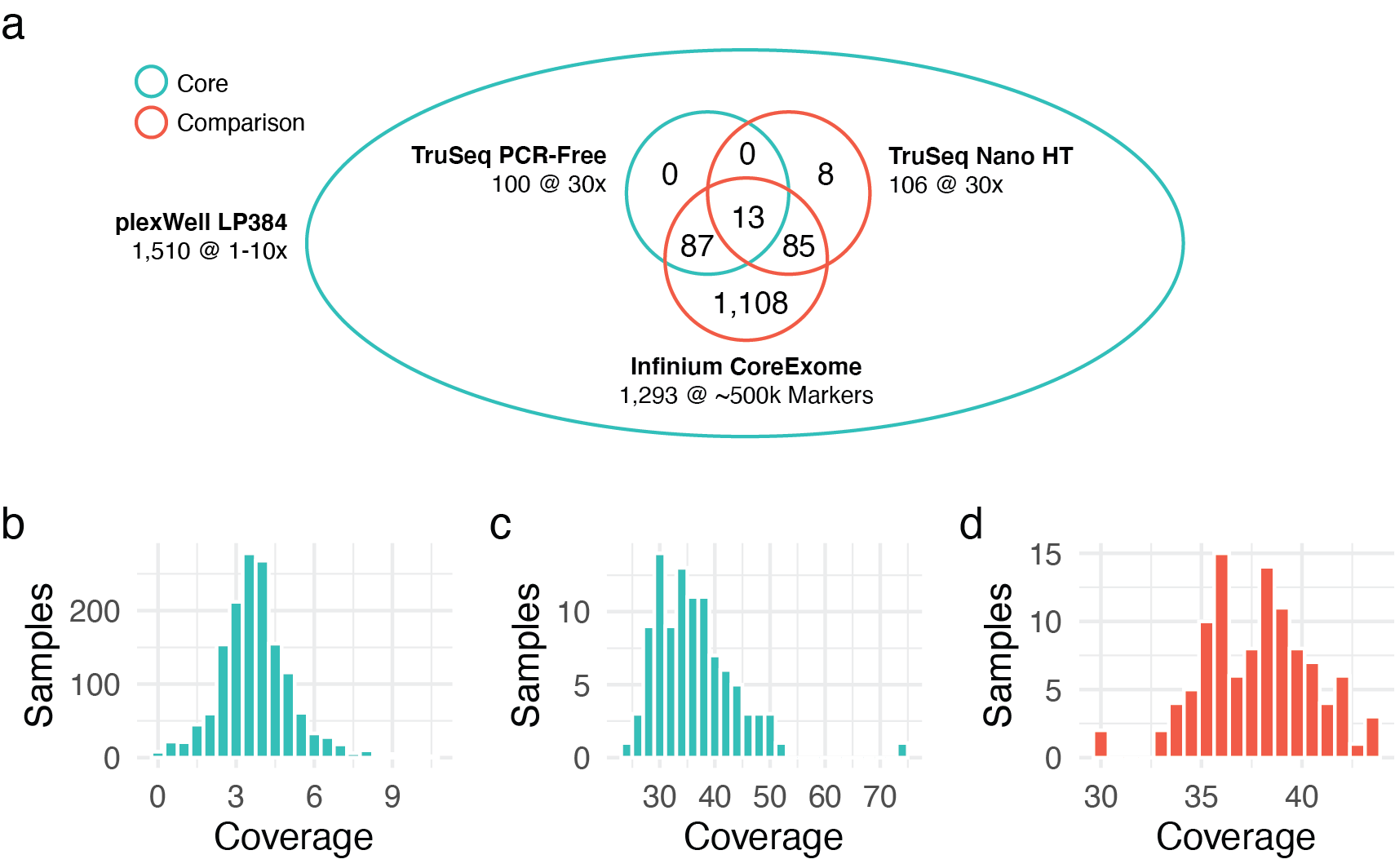


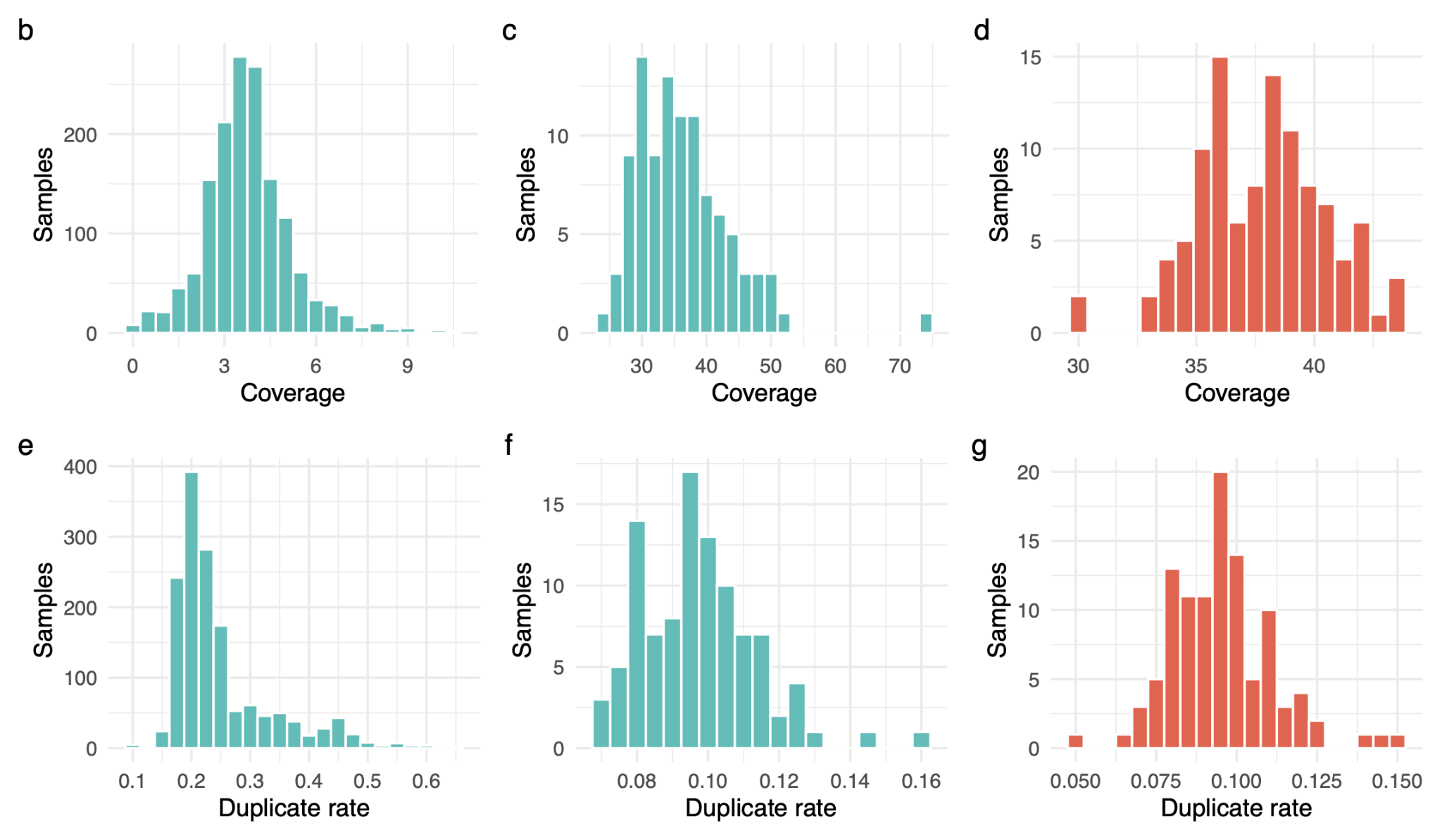


h

| ***Ethnicity*** | ***plexWell 4x*** | ***TruSeq PCR-free 30x*** | ***TruSeq Nano 30x*** |
| --- | --- | --- | --- |
| Aotearoa New Zealand Māori | 716 | 51 | 66 |
| Samoan | 201 | 14 | 1 |
| Mixed Polynesian | 176 | 12 | 5 |
| Cook Island Māori | 118 | 9 | 21 |
| Tongan | 97 | 6 | 0 |
| Pukapukan | 65 | 4 | 0 |
| Niuean | 28 | 2 | 0 |
| Tuvaluan | 4 | 1 | 0 |
| Tokelauan | 2 | 0 | 0 |
| European | 2 | 1 | 0 |
| Tahitian | 1 | 0 | 0 |

**Figure S2.** a) Overview of data types available for participants and how they overlap. b) Distribution of de-duplicated sequencing coverage per sample for low-pass samples, c) TruSeq PCR-free high-coverage samples, d) TruSeq Nano high-coverage samples. e) Distribution of sequencing duplicate rates per sample for low-pass samples, f) for TruSeq PCR-free samples, and g) for TruSeq Nano samples. h) Breakdown of number of individuals by self-reported ethnicity and sequencing type.

**S3. Pipeline Optimization: Effect of GQ Filtering on Indels**


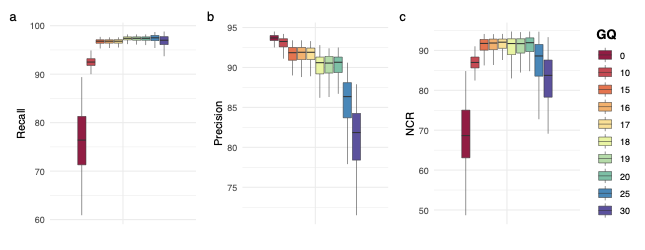


**Figure S3.** Effect of GQ filtering on indel calling performance. a) Recall, b) Precision, and c) NCR for indels over varying GQ thresholds.

**S4. Pipeline Optimization: Performance of Flagged Sites**


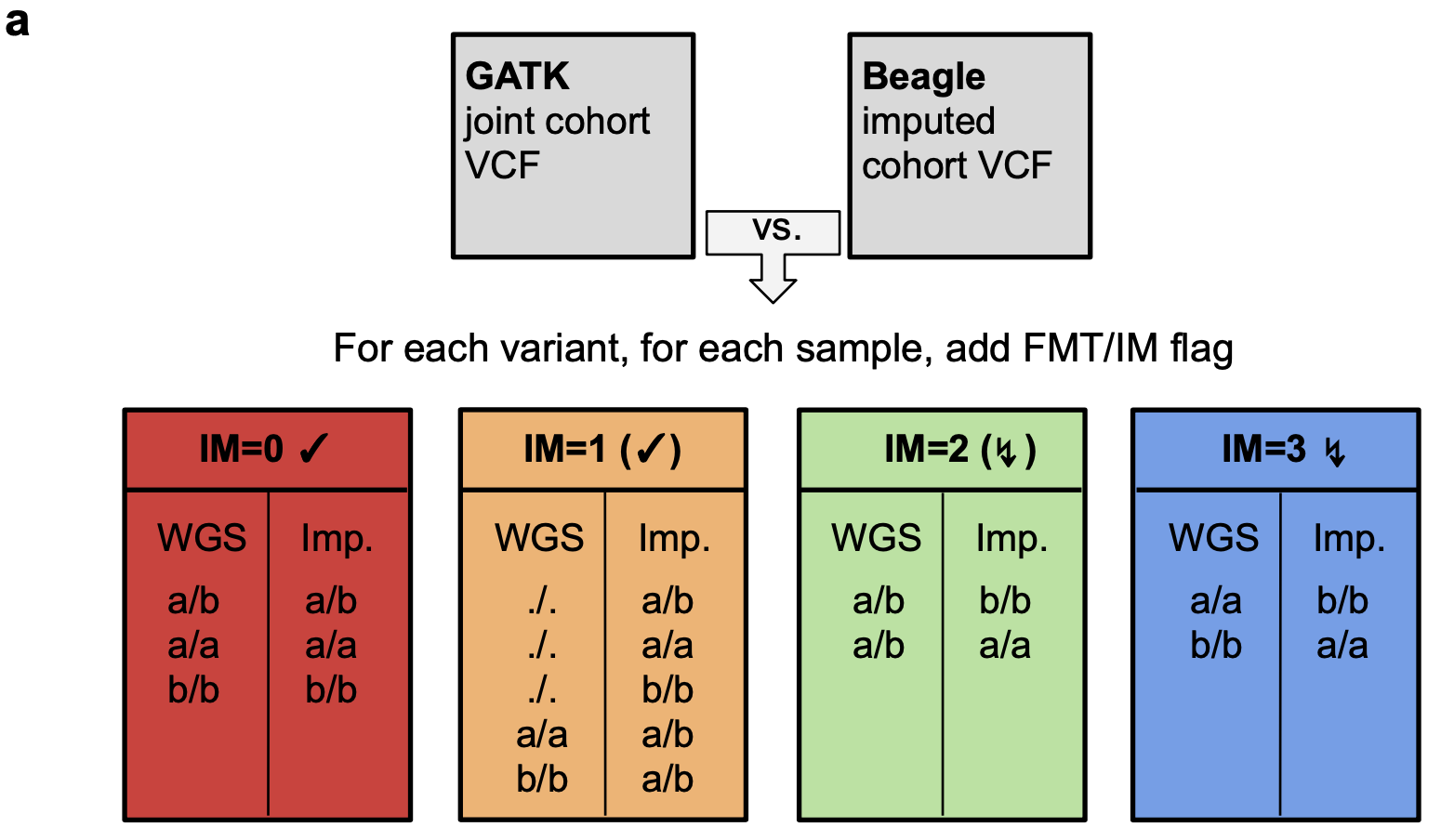

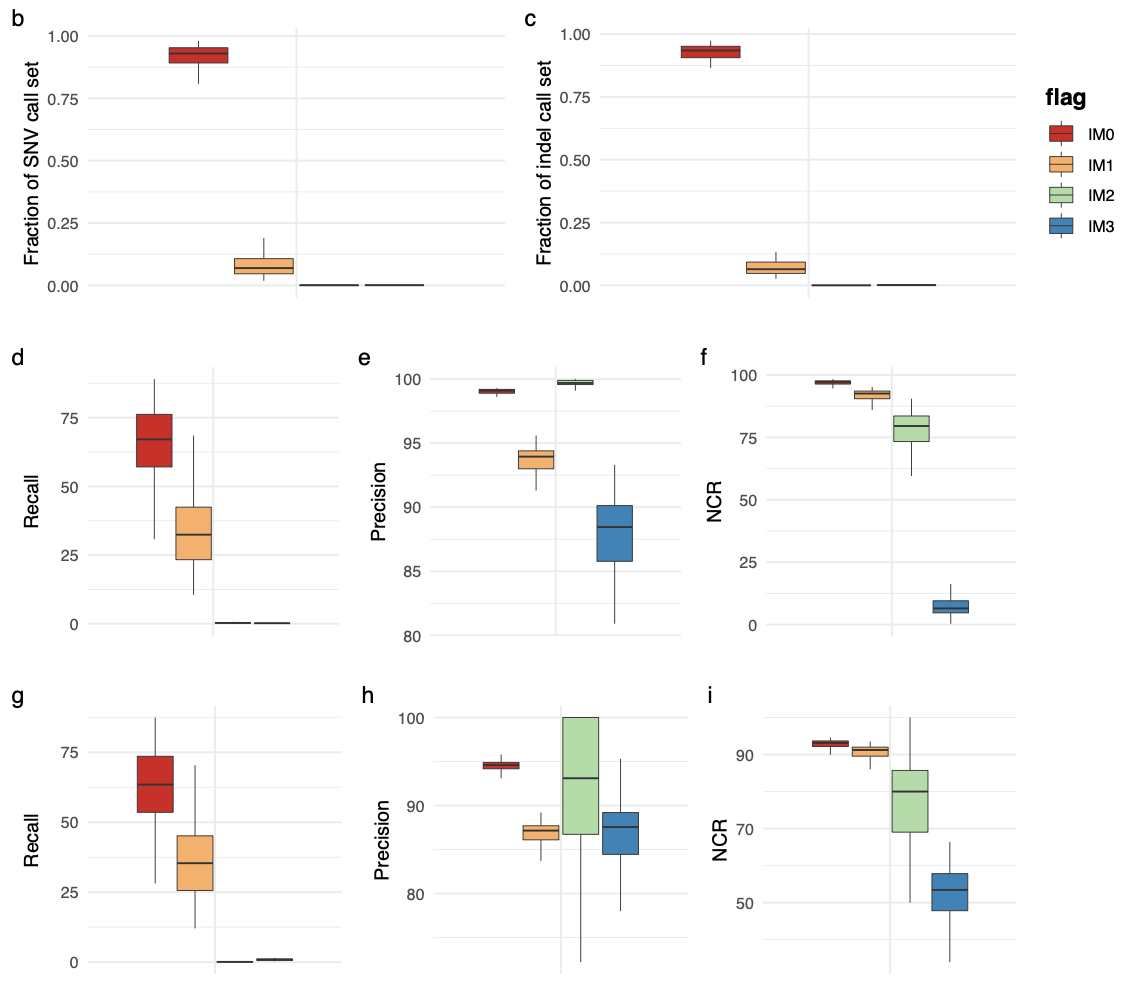


**Figure S4.** Accuracy of flagged sites by flag type. a) Overview of the different flag types that characterize variants by comparing (filtered) sequencing-based genotype with genotype after imputation. A call is flagged with IM=0 if sequencing-based genotype and imputed genotype agree fully. Given low coverage, we consider the lack of sequencing data evidence for an imputed allele as “not inconsistent” while the disappearance of an allele after imputation is categorized as “inconsistent”. IM=1 therefore flags imputed calls that are not inconsistent with the sequencing-based call (either because it was missing or we may have only observed one of two alleles in sequencing). IM=2 and IM=3 flag sites that are inconsistent between sequencing-based and imputed calls, where IM=2 calls were heterozygous in sequencing (potentially due to sequencing or mapping artifacts or contamination, or an error in imputation) and IM=3 calls were homozygous for the opposite allele. b) Fraction of SNV calls in each IM flag category. c) Fraction of indel calls in each IM flag category. d) Recall (normalized to each individual’s overall SNV recall), e) Precision, and f) NCR of SNVs. g) Normalized recall, h) Precision, and i) NCR of indels.

**S5. Detailed Performance of SNV & Indel Calling over Coverage**


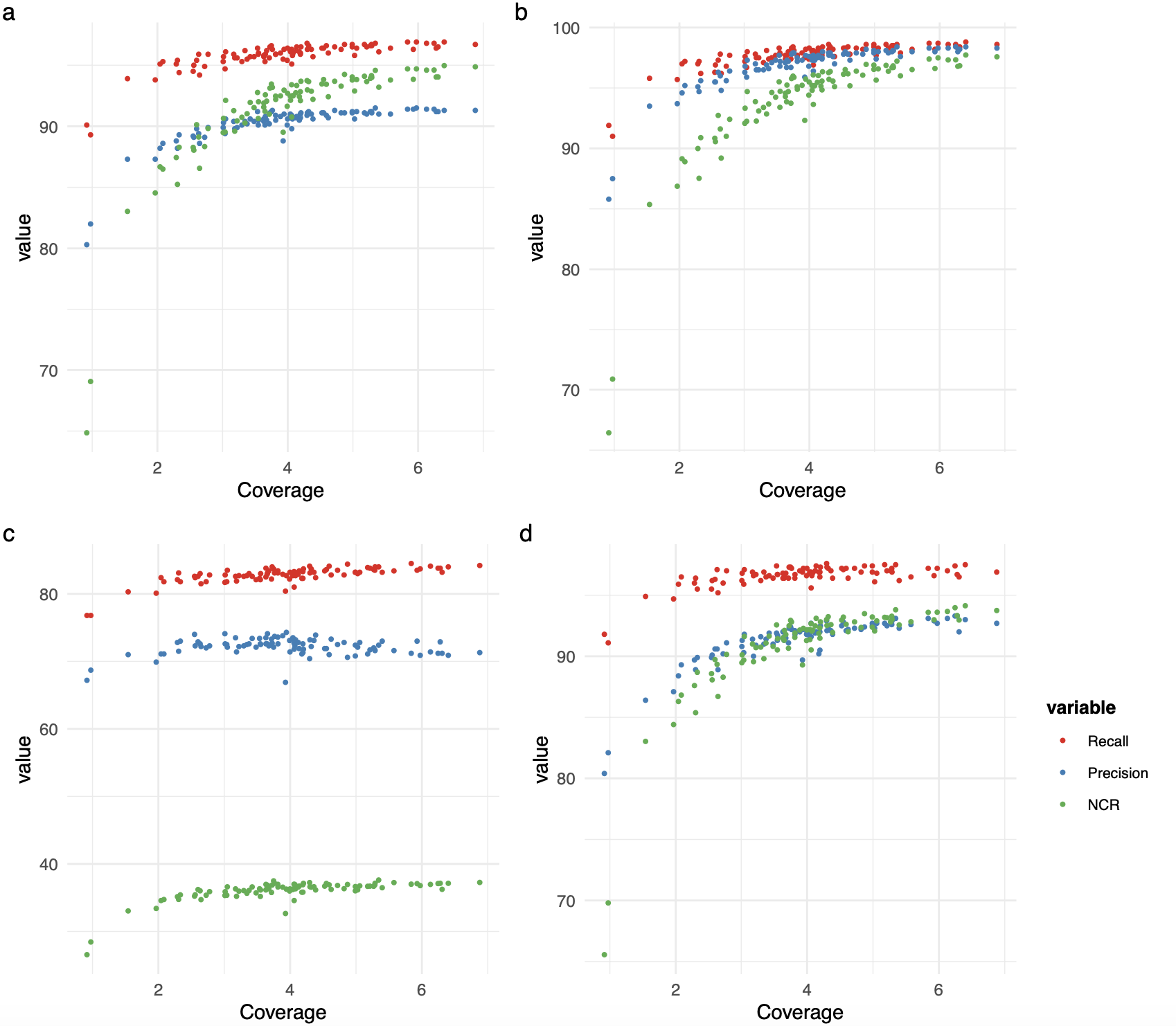


**Figure S5.** Detailed performance (recall, precision, and NCR) of SNV and indel calling both genomewide (including repetitive regions) as well as in high-confidence regions only, shown over coverage. a) SNVs genomewide, b) SNVs in high-confidence regions, c) Indels genomewide, d) Indels in high-confidence regions.

**S6. Effect of Joint Variant Calling and Inclusion of High-coverage Data**

**
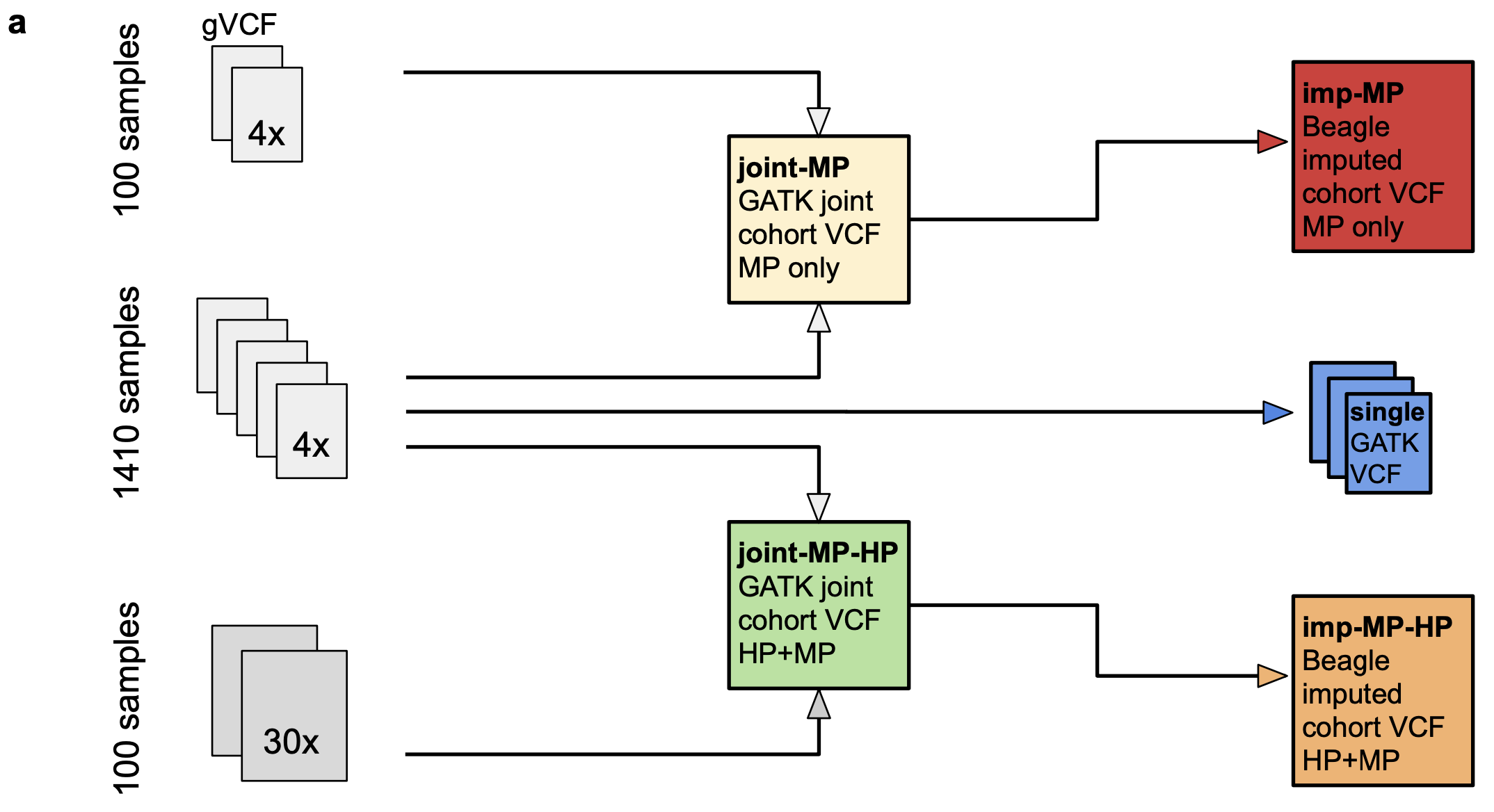
**


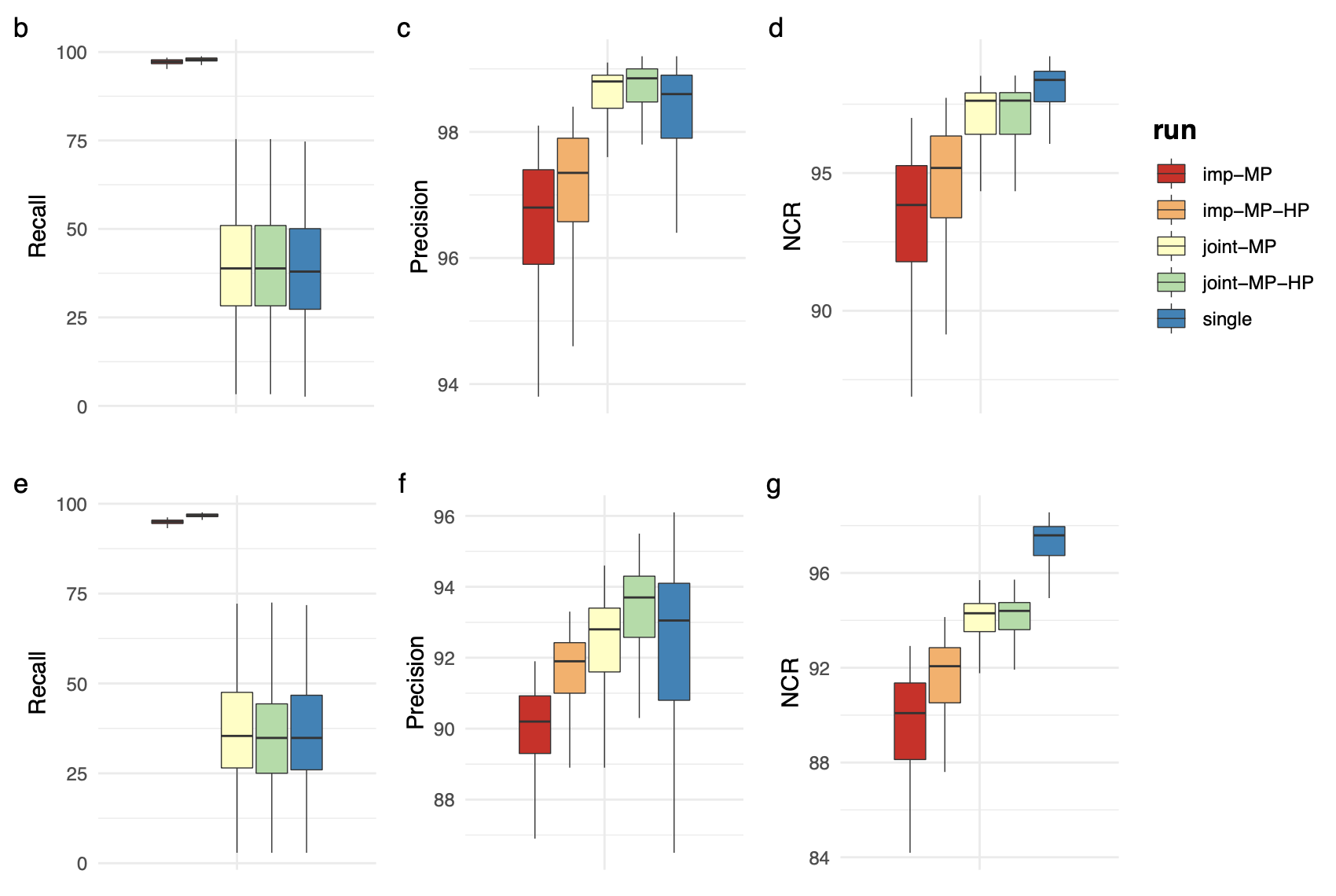


**Figure S6.** Performance comparison across different pipeline stages/runs. a) Overview of tested call sets. “Single” refers to individually called mid-pass data (GQ>17). “MP” and “MP-HP” refer to the joint-called (“joint”) and imputed (“imp”) call sets using mid-pass data from 1510 individuals (MP) and mid-pass data from 1410 individuals plus high-pass data from 100 high-pass individuals (MP-HP) For more details see methods. b) Recall, c) Precision, and d) NCR for SNVs. e) Recall, f) Precision, and g) NCR for indels.

**S7. European Admixture Analysis**

**
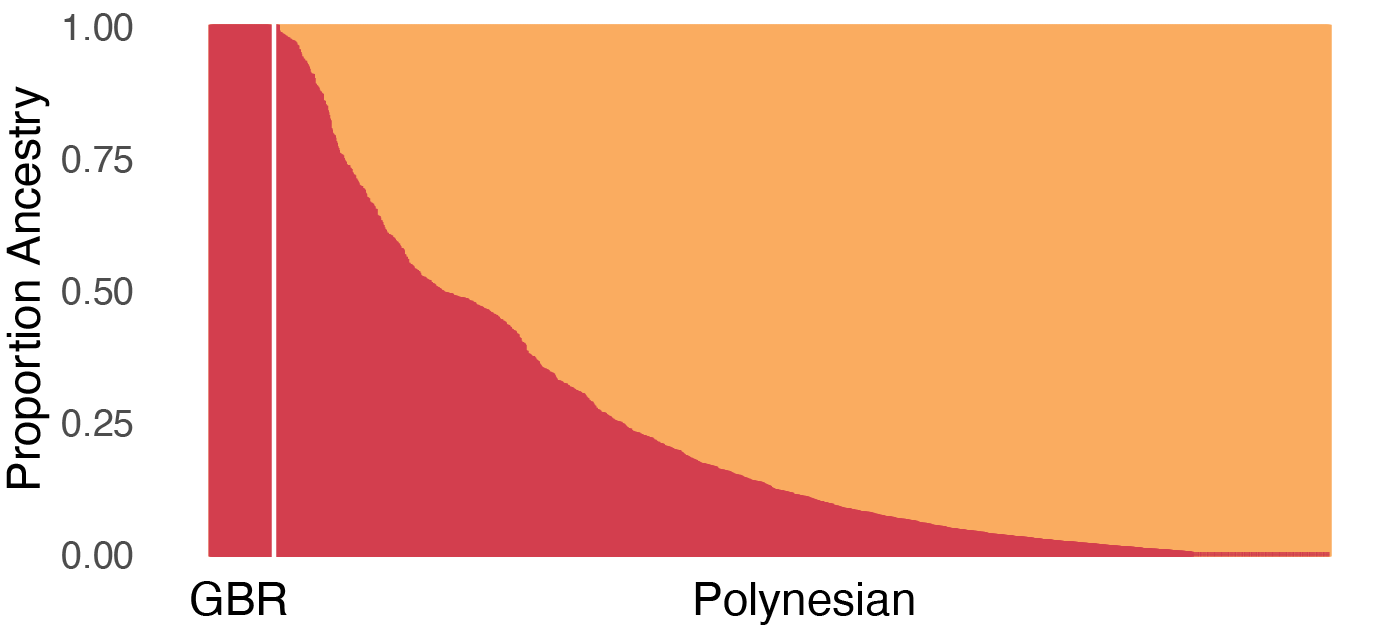
**

**Figure S7.** Analysis of European admixture in the study cohort. ADMIXTURE was run assuming two populations on the cohort with 91 British individuals from 1000 Genomes (GBR) included to capture European ancestry. Shown are the proportions of ancestry estimated (population 1 = red, population 2 = orange). Individuals are ordered by cohort (GBR/Polynesian). Analysis of PC1 from PC analysis versus proportion of population 1 ancestry from ADMIXTURE analysis found that PC1 is highly

correlated with the degree of estimated European ancestry (Spearman’s ρ = -0.89, p < 2.2e-16).

**S8. Principal Component Analysis**


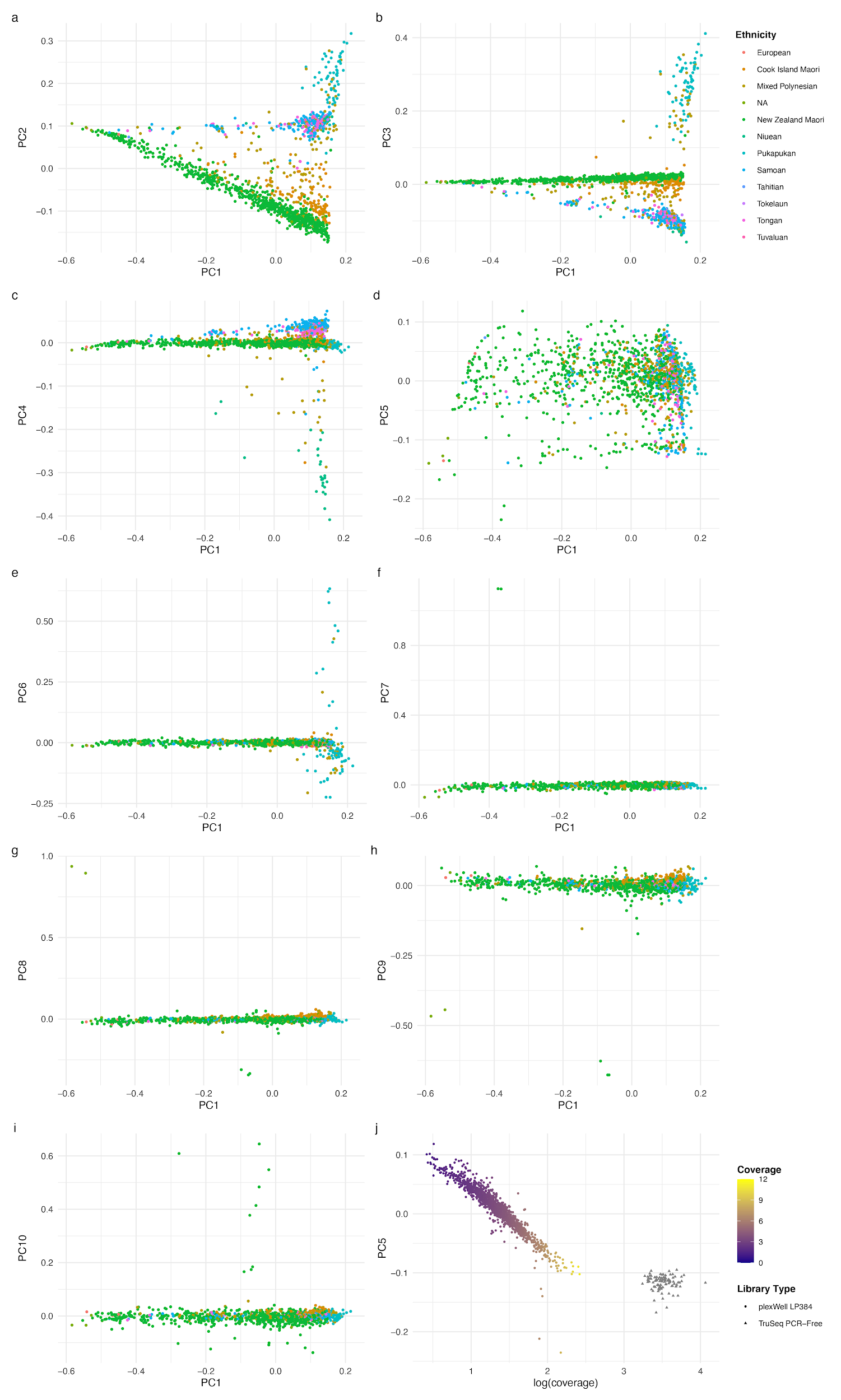


**Figure S8.** Principal component (PC) analysis of imputed genotype calls. a-i) PC1 vs PC2-10, labeled based on self-reported ethnicity. j) PC5 vs log(coverage) with data points colored by sequencing depth and symbols corresponding to library type (plexWell LP384 used for low-pass sequencing and TrueSeq PCR-Free used for high-pass sequencing). Individuals with ≥ 1.5x coverage and both SNVs and indels in high-confidence regions of the genome were used for the analyses.

**S9. Effect of Cohort Size on Variant Calling and Imputation**


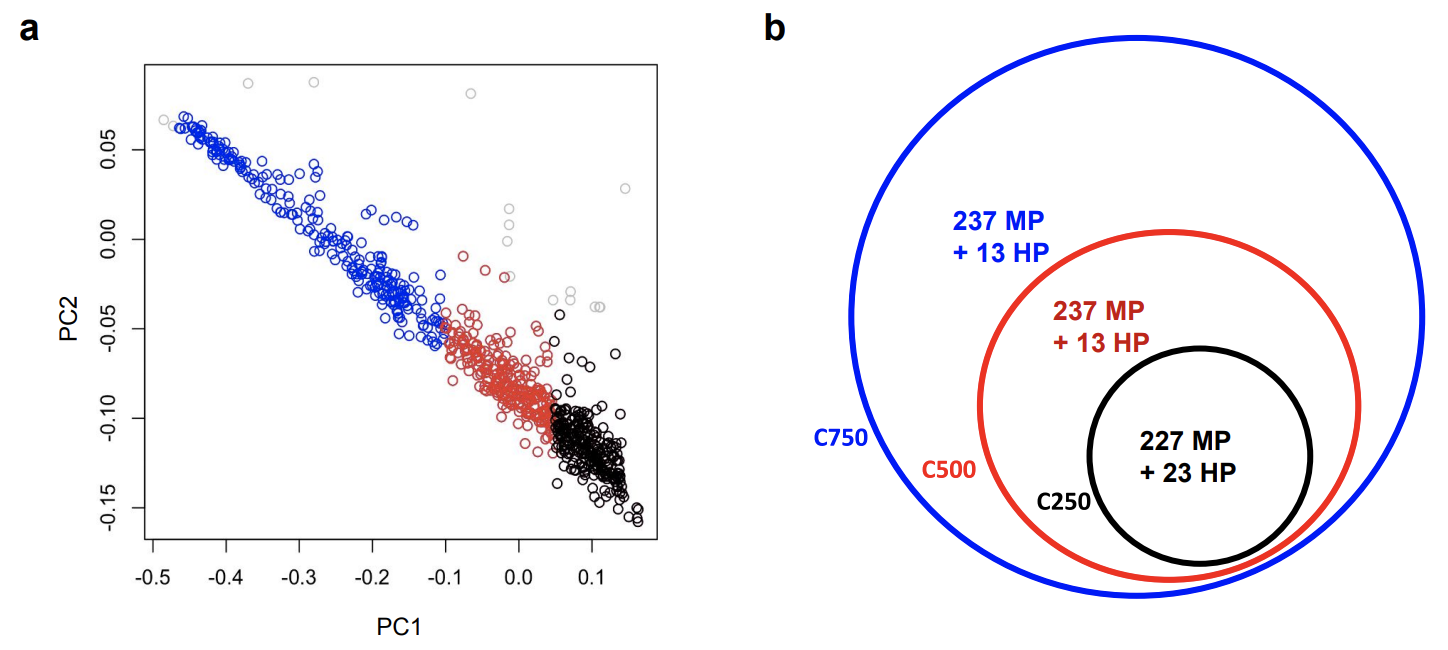


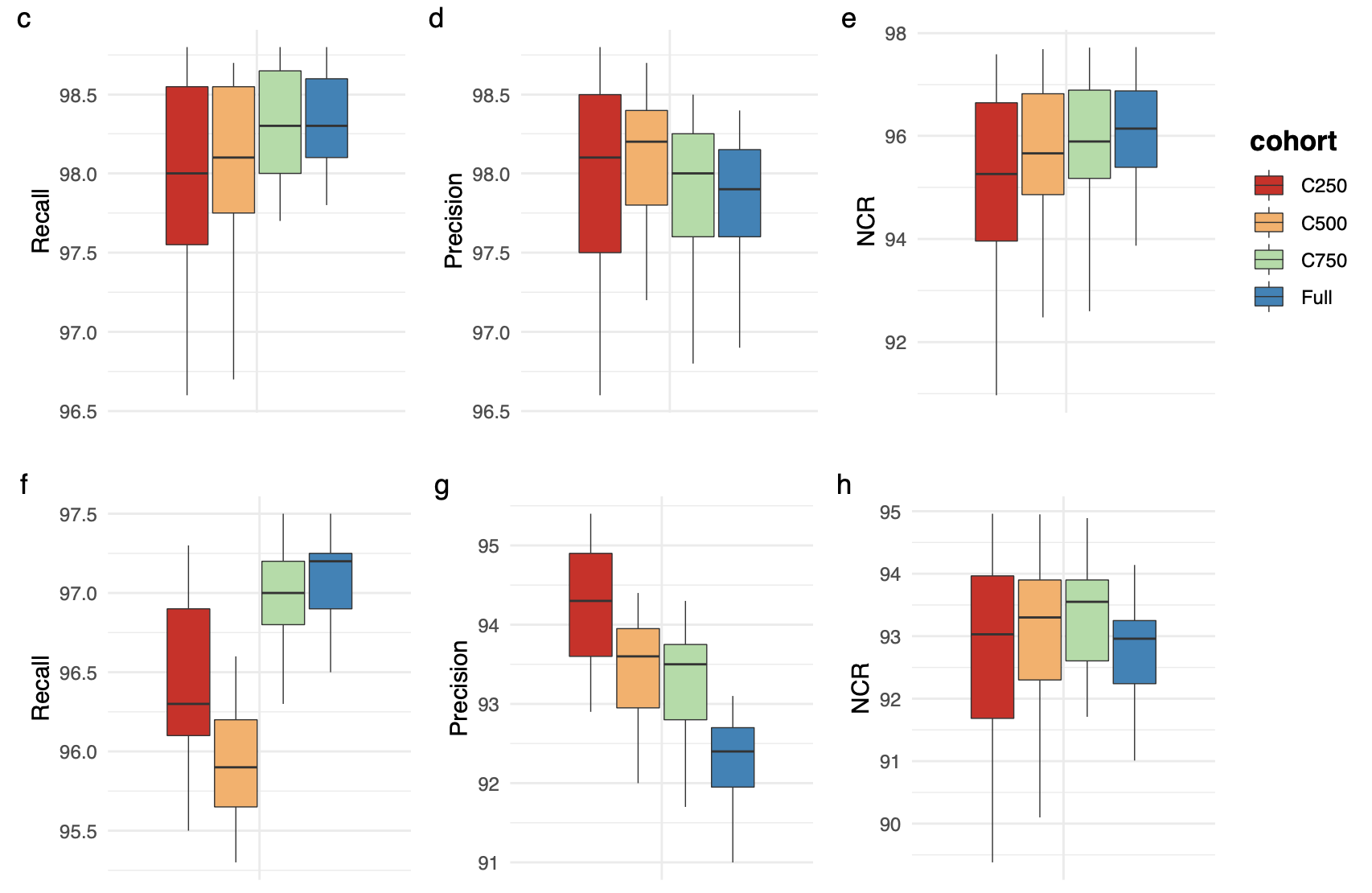


**Figure S9.** Effect of cohort size on performance. a) PCA of self-reported Aotearoa New Zealand Māori individuals that were included in the analysis. b) Sequencing type breakdown within subcohorts (MP, mid-pass, HP, high-pass). c) Recall, d) Precision, and e) NCR for SNVs. f) Recall, g) Precision, and h) NCR for indels.

**S10. Performance Comparison with Array Data Imputed into 1000 Genomes**

**
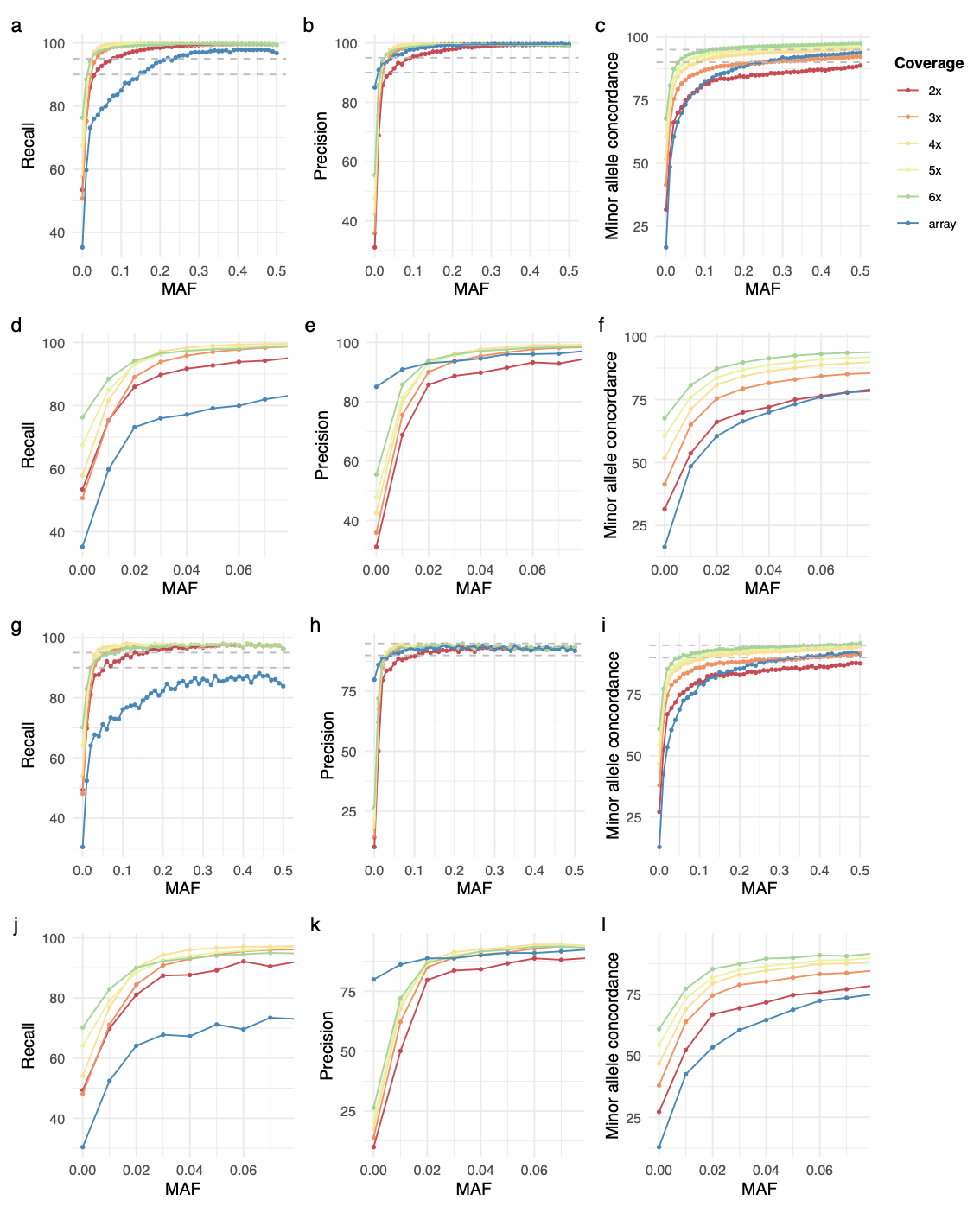
**

**Figure S10.** MAF-based comparison of variants in high-confidence regions, split by coverage level. a) Recall, b) Precision, and c) NCR of SNVs over the full MAF range. Panels d), e), and f) show the same plots zoomed in on the 0-7.5% MAF range. Panels g) to l) show the same for indels.

**S11. Allele Frequency Distribution of Rare and Novel Variants**


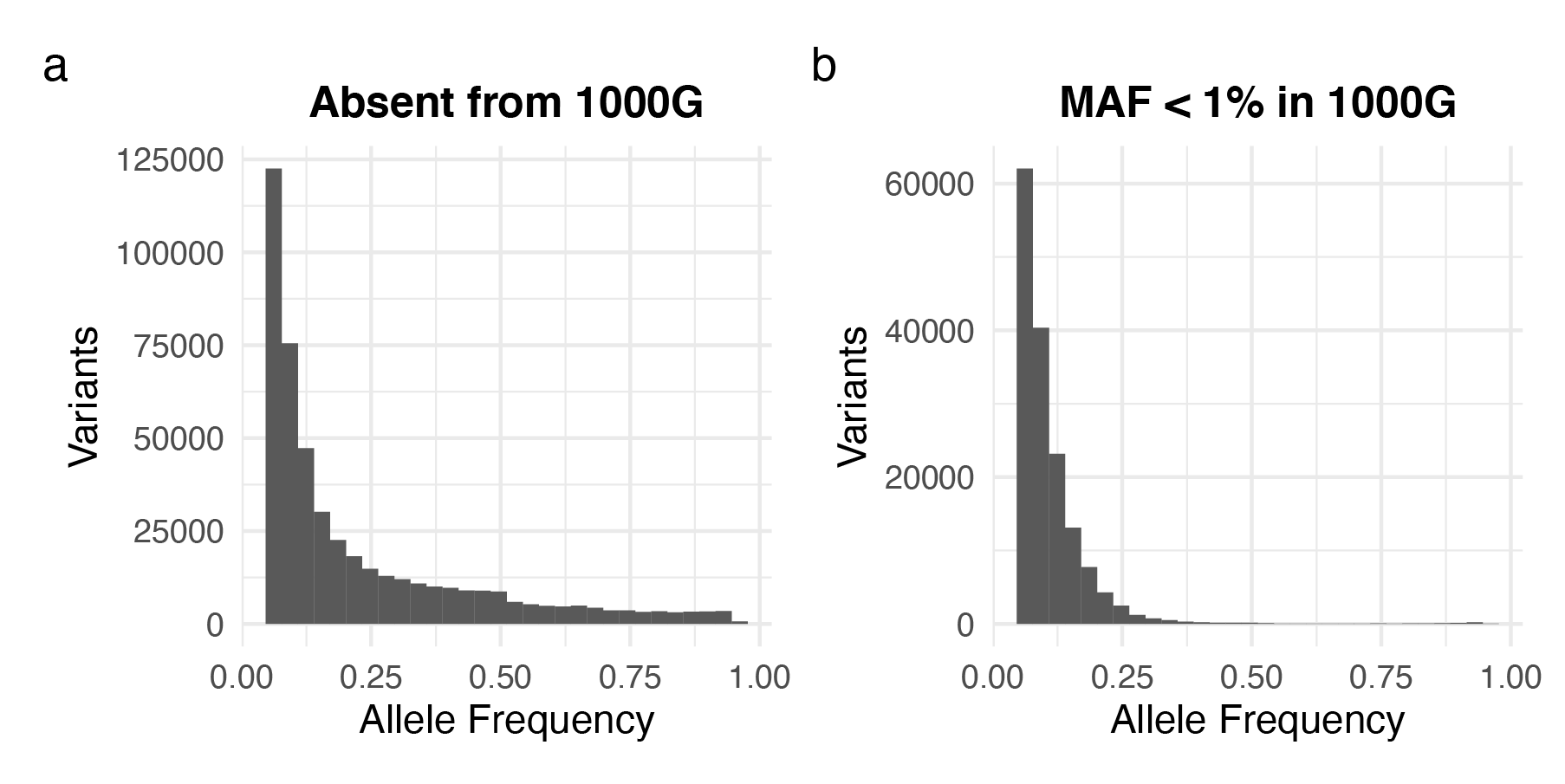


**Figure S11.** Allele frequency distribution of common (MAF > 5%) variants in the study cohort that are either absent from (a) or rare in (b) 1000 Genomes. Indels located in high-confidence regions of the genome and all SNVs were included in the analysis.
